# Gene expression profiling of mouse p53-deficient epidermal carcinoma defines molecular determinants of human cancer malignancy

**DOI:** 10.1186/1476-4598-9-193

**Published:** 2010-07-14

**Authors:** Ramón García-Escudero, Ana B Martínez-Cruz, Mirentxu Santos, Corina Lorz, Carmen Segrelles, Guillermo Garaulet, Cristina Saiz-Ladera, Clotilde Costa, Águeda Buitrago-Pérez, Marta Dueñas, Jesús M Paramio

**Affiliations:** 1 Molecular Oncology Unit, Division of Biomedicine, CIEMAT, Ave. Complutense 22, E-28040 Madrid, Spain

## Abstract

**Background:**

The epidermal specific ablation of *Trp53 *gene leads to the spontaneous development of aggressive tumors in mice through a process that is accelerated by the simultaneous ablation of *Rb *gene. Since alterations of p53-dependent pathway are common hallmarks of aggressive, poor prognostic human cancers, these mouse models can recapitulate the molecular features of some of these human malignancies.

**Results:**

To evaluate this possibility, gene expression microarray analysis was performed in mouse samples. The mouse tumors display increased expression of cell cycle and chromosomal instability associated genes. Remarkably, they are also enriched in human embryonic stem cell gene signatures, a characteristic feature of human aggressive tumors. Using cross-species comparison and meta-analytical approaches, we also observed that spontaneous mouse tumors display robust similarities with gene expression profiles of human tumors bearing mutated TP53, or displaying poor prognostic outcome, from multiple body tissues. We have obtained a 20-gene signature whose genes are overexpressed in mouse tumors and can identify human tumors with poor outcome from breast cancer, astrocytoma and multiple myeloma. This signature was consistently overexpressed in additional mouse tumors using microarray analysis. Two of the genes of this signature, AURKA and UBE2C, were validated in human breast and cervical cancer as potential biomarkers of malignancy.

**Conclusions:**

Our analyses demonstrate that these mouse models are promising preclinical tools aimed to search for malignancy biomarkers and to test targeted therapies of prospective use in human aggressive tumors and/or with p53 mutation or inactivation.

## Introduction

Mouse models of human cancer have become essential tools for preclinical analysis of antitumoral drug discovery. To demonstrate that these models faithfully recapitulate human disease, a deep characterization of the tumors is required. Functional comparative genomics is one of the most powerful techniques for such validation. Moreover, such analyses have also evidenced that mouse models display the complexity of human cancer genomes. Cross-species studies using genomic-based technologies have indicated the preservation of oncogene transcriptional signatures [1,2] or the synteny of tumor-associated copy number alterations [3-5]. Furthermore, comparison between mouse and human samples have demonstrated the conservation of somatic signature mutational events [4,5], and have enabled the efficient identification of new oncogenes in human cancers [6].

The p53 protein is a transcription factor that responds to diverse stress signals (including DNA damage, oncogene activation and various metabolic limitations) to regulate many target genes that induce cell-cycle arrest, apoptosis, senescence, autophagy, DNA repair and/or metabolic changes [7,8]. As a consequence, the p53 pathway is a crucial mechanism for effective tumor suppression. Somatic or germline mutations in TP53 gene that compromise its function occur in around 50% of all human cancers (IARC TP53 mutation database, version R14, November 2009 is the latest, [9]), and even those tumors that retain wild-type p53 frequently show defects in the pathways leading to its functional inactivation [10], such as amplification of MDM2 [11]. Furthermore, somatic mutations in TP53 have been associated with poor outcome in most human cancers [9,11]. Importantly, both somatic and germline TP53 mutations are usually followed by loss of heterozygosity (LOH) during tumor progression [12], which suggest that a selective force inactivates the remaining wild-type allele. The majority of TP53 mutations are missense (73.6%), and many of these missense mutant p53 forms not only lose their tumor suppressive function and acquire dominant-negative activities, but also gain new oncogenic properties that are independent of wild-type p53, the so called gain-of-function mutants [12]. However, an important proportion of mutations would give rise to a truncated p53 protein, such as nonsense, frameshift and large deletion mutations (16.6% of all mutations). The essential role of p53 in tumor suppression has also been demonstrated using genetically modified mice, whereby *Trp53 *deletion or missense mutations induce tumor formation in multiple tissues and organs [13]. We and others have reported that the somatic inactivation of p53 tumor suppressor in stratified epithelia, using the Cre-LoxP system (hereafter Trp53^ΔEC^), induces spontaneous development of skin squamous cell carcinoma (SCC) [14,15]. Besides, skin tumor development is accelerated by inactivation of both *Trp53 *and *Rb *genes (hereafter Rb^ΔEC^; Trp53^ΔEC^) [15]. Interestingly, tumors arising in both genotypes, which originate in close proximity to hair bulge, where the adult epidermal stem cells reside, display high aggressive characteristics including premature epithelial-mesenchymal transition (EMT) and distant metastasis (manuscript in preparation).

Here we have characterized the differential gene expression patterns between tumor and normal skin tissue, in order to obtain putative target genes for antitumoral therapies and/or for biomarker discovery. We have observed that primary tumors from Trp53^ΔEC ^and Rb^ΔEC^; Trp53^ΔEC ^show a predominant overexpression of genes involved in cell cycle progression and mitosis regulation. The mouse tumors also display a core transcriptional profile similar to human embryonic stem cells, a feature associated with increased aggressiveness of human tumors. Cross-species studies demonstrate that the overexpressed genes could significantly identify human cancers bearing p53-mutations and/or highly aggressive behavior. Collectively, we have obtained a set of genes with reproducible overexpression in mouse samples, which could be used as targets for preclinical antitumor therapies and as biomarkers of malignancy in primary tumors.

## Results

Inactivation of *Trp53 *in stratified epithelia leads to the generation of spontaneous epidermal tumors with a complete penetrance by one year of age [15]. The simultaneous inactivation of *Rb1 *and *Trp53 *leads to earlier appearance of the tumors and faster growth at early stages when compared to inactivation of only *Trp53 *alleles. To fully characterize the molecular features of these tumors we performed gene expression profiling using Affymetrix microarrays using total RNA from 27 carcinomas arising in Trp53^ΔEC ^and Rb^ΔEC^; Trp53^ΔEC ^mice, and 9 normal, wild type skin samples.

### Gene expression comparison of tumours arising in Trp53^ΔEC ^and Rb^ΔEC^; Trp53^ΔEC ^mouse models

**Figure 1:**
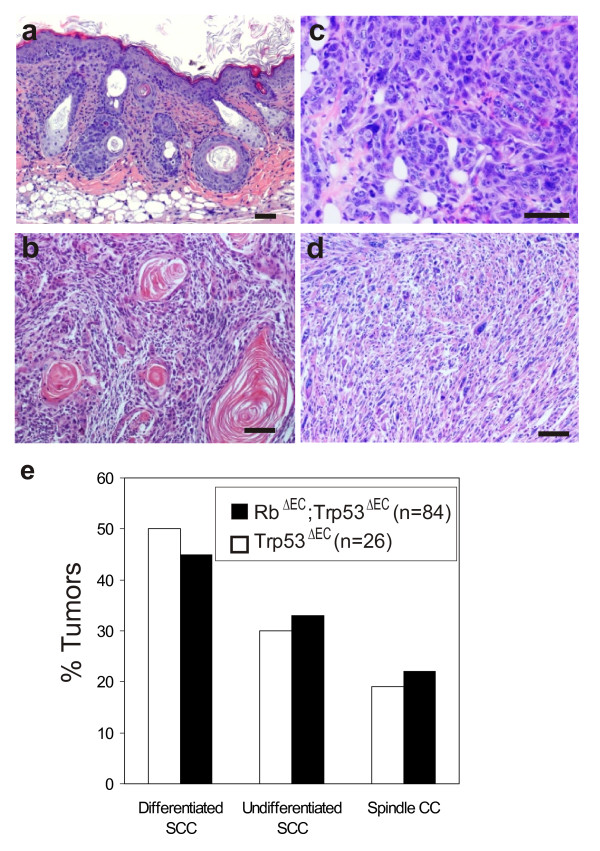
**Histological analysis of tumors arising in Trp53^ΔEC ^and Rb^ΔEC^; Trp53^ΔEC ^mice**. Histology sections showing (**a**) a representative example of subcutaneous tumor mass arising from the hair follicle in a Rb^ΔEC^; Trp53^ΔEC ^mouse, (**b**) a poorly differentiated SCC from a Rb^ΔEC^; Trp53^ΔEC ^mouse, (**c**) a highly undifferentiated tumor from a Trp53^ΔEC ^mouse, and (**d**) a spindle cell carcinoma in a Rb^ΔEC^; Trp53^ΔEC ^mouse. (**e**) Proportion of tumors arising in both mouse models. Advanced stage tumors were classified histopathologically into poorly differentiated, undifferentiated and spindle cell carcinomas.

In both genotypes the tumors appeared as small subcutaneous squamous lesions originating in or close to the hair follicles (Fig. 1a). They exhibit a fast growth leading to poorly differentiated squamous cell carcinomas (Fig. 1b), which rapidly progress, lose the expression of differentiation markers such as K10, K6 and K17 [15], and become highly undifferentiated carcinomas (Fig. 1c) and in, some cases evolve to spindle cell carcinomas (Fig. 1d), possibly by means of a premature EMT process. Overall, at the most advanced stage Trp53^ΔEC ^and Rb^ΔEC^; Trp53^ΔEC ^mice tumors are very similar and histopathologically indistinguishable (Fig. 1e) [15].

**Figure 2:**
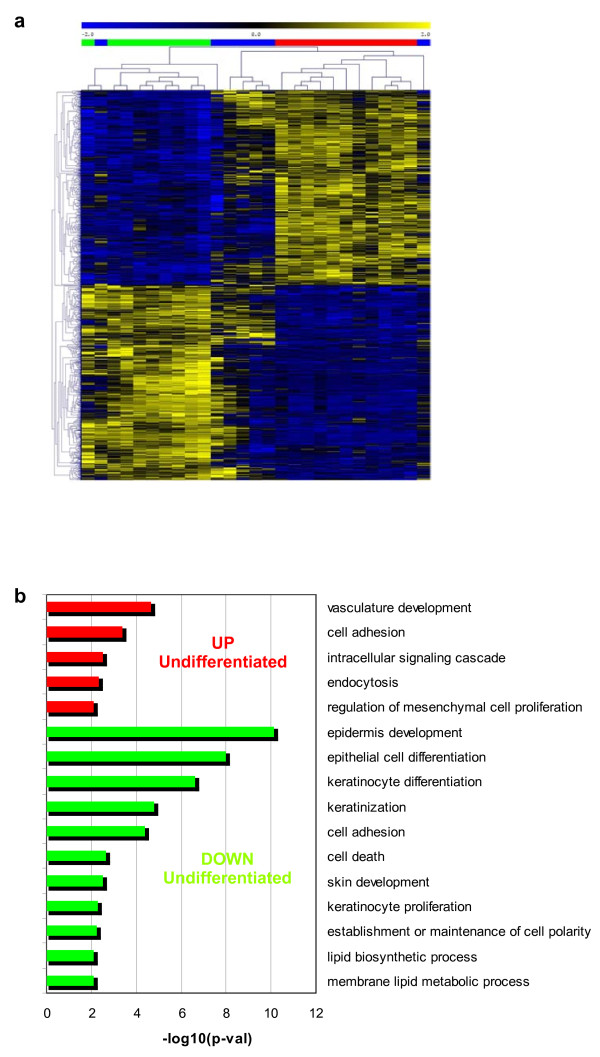
**Gene expression differences of Trp53^ΔEC ^and Rb^ΔEC^; Trp53^ΔEC ^mouse tumors based on histological grade**. Gene expression deregulation related to carcinoma differentiation was done using supervised Ttest analysis between poorly differentiated carcinomas and undifferentiated/spindle carcinomas. Results are shown for 2% of probesets overexpressed (n = 902, FDR corrected p-val = 0.002) or underexpressed (n = 902, FDR corrected p-val = 0.001) in undifferentiated/spindle carcinomas. (**a**) Hierarchical clustering analysis using euclidean distance and average linkage clustering of probesets and all carcinomas: poorly differentiated (green), undifferentiated/spindle (red), and mixed, containing differentiated and undifferentiated areas (blue). (**b**) Enrichment analysis in GO biological processes of the deregulated genes evidenced overexpression of genes involved in vasculature development, cell adhesion, and endocytosis (red bars), and underexpression of genes involved in keratinocyte differentiation or cell death (green bars) in undifferentiated/spindle carcinomas. p-val: significance of enrichment.

In order to characterize the tumor progression and to compare tumors with different histological grade at the molecular level, we performed supervised analysis of differential gene expression. This analysis showed significant differences depending on tumor histological grade (undifferentiated/spindle vs. poorly differentiated carcinomas) (Fig. 2). Enrichment analysis of Gene Ontology Biological Processes (GOBP) terms demonstrated increased expression of genes involved in vasculature development, cell adhesion, and endocytosis in undifferentiated/spindle carcinomas with respect to poorly differentiated carcinomas. More specifically, we have found overexpression of genes that mediate EMT such as *Snai1*, *Zeb1 *or *Zeb2*, or genes associated with EMT such as *TgfbrII*, *Dab2*, *Vimentin*, *Col6a1 *and *Col6a2 *and *Adam19*. Also, we found in undifferentiated/spindle carcinomas a significant reduced expression of genes involved in keratinocyte and/or epidermal differentiation such as *Cdh1 *(*E-cadherin*), *Krt17*, *desmocollin 1*, *2 *and *3*, *desmoplakin*, *claudin 1*, *4 *and *8*, *Lama5*, *plakophilin 1 *and *3*, or *plakoglobin*. Some of these genes can be repressed in EMT by *Snai1*, *Zeb1 *or *Zeb2 *transcription factors [16]. The results confirm that the undifferentiated/spindle carcinomas display molecular features of EMT tumors.

The comparative analysis of tumor appearance in Trp53^ΔEC ^and Rb^ΔEC^; Trp53^ΔEC ^mice have revealed an accelerated tumor onset in Rb^ΔEC^; Trp53^ΔEC ^indicating the existence of cooperative functions for these tumor suppressors in epidermis, which is in contrast with the absence of spontaneous tumors [17] and the reduced susceptibility to chemical carcinogenesis in mice lacking epidermal pRb [18]. However, at the advanced stage there are no overt differences in differentiation, grade or growth rate between the two genotypes [15]. In order to identify possible molecular differences/similarities in the tumors arising in both mouse models, we also performed a supervised analysis of differential expression based on mouse genotype (Trp53^ΔEC ^*vs *Rb^ΔEC^; Trp53^ΔEC^). This analysis revealed that tumors in both genotypes are very similar, as only 83 probesets were differentially expressed at the significance level of FDR < 0.1 (Additional file 1). The result might indicate that although *Rb *somatic inactivation in double deficient mice accelerated the appearance and initial growth of tumors, it does not importantly contribute to the overall gene expression pattern of overt primary tumors.

### Trp53^ΔEC ^and Rb^ΔEC^; Trp53^ΔEC ^mouse tumors are enriched in human stem cell genes

Using gene enrichment analysis of 13 partially overlapping gene signatures that were compiled from the literature, Ben-Porath *et al. *reported that high grade, metastatic human tumors displayed gene expression programs similar to those described for human embryonic stem (ES) cells, and are also enriched for targets of key regulators of ES cell identity (Oct4, Sox2, and Nanog) and targets of Myc oncogene (key regulator of cell differentiation) [19]. On the contrary, these ES-like human tumor samples displayed down-regulation of genes bound by the Polycomb repressive complex 2 (PRC2) [19]. Ben Porath *et al. *study represents an important evidence of the similarities between gene expression programs of metastatic tumors and ES cells.

Spontaneous tumors arising in Trp53^ΔEC ^and Rb^ΔEC^; Trp53^ΔEC ^mice are high grade and aggressive, they originate from hair follicles (where adult epidermal stem cells reside) and, at early stages, display increased expression of certain epidermal stem cell markers such as keratin K15 [15]. Therefore, we wanted to analyze whether they also share gene expression patterns of human ES cells. To this, we downloaded the 13 gene signatures (described in Materials and Methods) used by Ben-Porath *et al*, and performed a similar analysis using Gene Set Enrichment Analysis (GSEA) [20,21] on the mouse samples. We observed that tumors are enriched in human ES cell genes, and in targets of Nanog, Sox2, and Myc transcription factors (Table 1). Conversely, mouse tumors displayed repression of Polycomb targets. The analysis demonstrates similar patterns of human ES cells gene programs within the mouse epidermal tumors from p53-deficient mouse, thus resembling most of the molecular features of high-grade, malignant human tumors.

**Table 1:** GSEA of human stem cell signatures

**Gene Set Name (N)**^**1**^	Number of enriched genes	NES	FDR q-val
ES EXP1 (315)	128	2.16	<0.0001*
ES EXP2 (32)	9	1.89	<0.0001*
MYC Targets2 (664)	182	1.63	0.0022*
SOX2 Targets (584)	166	1.54	0.0058*
MYC Targets1 (199)	55	1.47	0.013*
NANOG Targets (797)	225	1.38	0.021*
OCT4 Targets (242)	63	1.22	0.068
NOS Targets (152)	32	1.08	0.23
H3K27 BOUND (886)	245	-1.65	0.0026*
PRC2 Targets (508)	126	-1.59	0.0038*
EED Targets (800)	183	-1.55	0.0042*
SUZ12 Targets (819)	201	-1.65	0.005*
NOS TFS (32)	5	-1.08	0.24

^1 ^N: number of genes from each gene set in mouse chip.

NES: normalized enrichment score.

NES > 0: enrichment in tumors; NES < 0: enrichment in normal skin.

*Significant enrichment.

### Generation of a gene expression signature for epidermal tumors from p53-deficient mouse

Gene expression profiles comparing normal and tumoral samples provide information about genes that could display important functions in the carcinoma maintenance or aggressiveness, and non essential roles in the normal tissue. The therapeutic inhibition of these genes would not affect normal tissue homeostasis but may affect tumor growth or metastasis, thus becoming potential molecular targets for therapy. In addition, interspecies comparison between human and mouse could also be useful to determine which genes display similar expression patterns so they can be considered validated targets for therapy and/or biomarkers of human cancer. In order to identify such possible genes in our mouse tumor samples, we divided the full dataset in two datasets containing 20 and 16 samples selected randomly: i) a training dataset, with 5 normal skin and 15 tumors from both genotypes, which were used to compare with human tumors and to select gene targets/biomarkers; ii) a testing dataset, with 4 normal skin and 12 tumors from both genotypes, in order to validate the selected genes in new, external samples.

**Figure 3:**
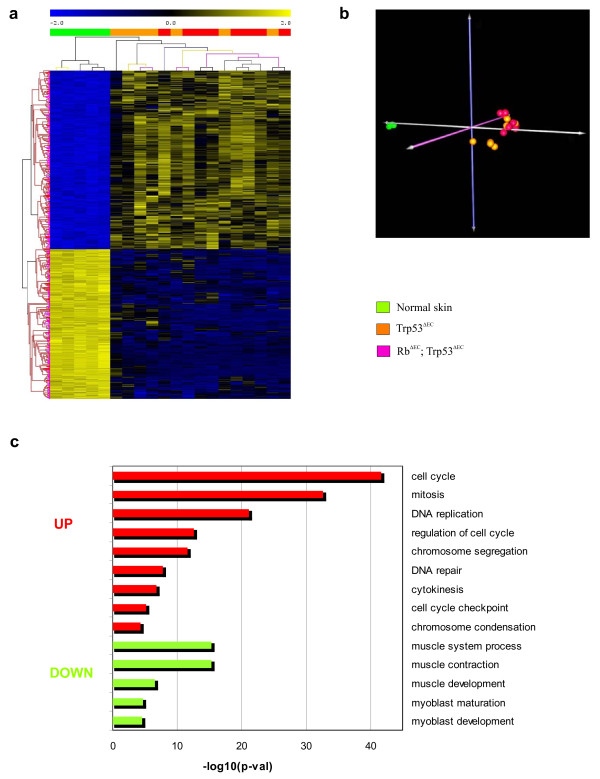
**Tumor signature of p53-deficient mouse**. (**a**) Unsupervised hierarchical clustering of skin tumors using the tumor signature of p53-deficient mouse was done with Pearson distance metrics (average linkage method), and the clustering was iterated 100 times using bootstrap resampling. Columns represent samples, and rows are genes. Green samples are normal control skin from adult mice. Orange samples are skin tumors from Trp53^ΔEC ^genotype, and red samples from Rb^ΔEC^; Trp53^ΔEC^. (**b**) Principal Component Analysis of animal samples using the tumor signature of p53-deficient mouse. Normal skin and mouse tumors are clearly separated along the principal component 1 (PC1), demonstrating that the signature clearly distinguishes between normal and cancer tissues. Moreover, tumors arising from either genotype (Trp53^ΔEC ^or Rb^ΔEC^; Trp53^ΔEC^) cluster in the same area, showing that the signature is common for them. Green samples are normal skin from adult mice. Orange samples are skin tumors from Trp53^ΔEC ^genotype, and red samples from Rb^ΔEC^; Trp53^ΔEC^. (**c**) Enrichment analysis in GO biological processes from the gene signature of p53-deficient mouse. Overexpressed genes are related with cell cycle, mitosis, or DNA repair (red bars). Downregulated genes are involved in muscle processes, probably due to loss of muscle tissue in aggressive tumors (green bars). p-val: significance of enrichment.

Differential expression analysis between mouse tumors and control normal skin in the training dataset provided a gene expression signature of 682-probesets (371 overexpressed and 311 underexpressed in tumors) (Additional files 2 and 3). Unsupervised hierarchical clustering analysis and principal component analysis of the samples using this gene signature revealed a high degree of similarity between tumors of both Trp53^ΔEC ^and Rb^ΔEC^; Trp53^ΔEC ^mouse genotypes (Fig. 3a and 3b), which is in line with the above results (see Additional file 1). Thus, the observed gene expression profile can be ascribed to a tumor gene signature from p53-deficient mouse, as somatic inactivation of *Trp53 *alleles is the common hallmark of both transgenic lines. Consistent with the functional roles of p53, most of the overexpressed genes in the tumors are involved in DNA replication and repair, or genomic instability and cell cycle checkpoint, as evidenced by enrichment analysis of GOBP terms (Fig. 3c). This finding is coincident with previous reports showing that TP53 mutations are associated with increased global genomic instability [22] and the observation of a high chromosomal instability in tumors and in pretumoral skin of Trp53^ΔEC ^mice [23]. In sharp contrast, downregulated genes are related to muscle development and physiology, that may be explained by the absence of dermal muscle layers in tumor samples (see also Fig. 1).

### Gene expression comparison between mouse and human tumors

**Figure 4:**
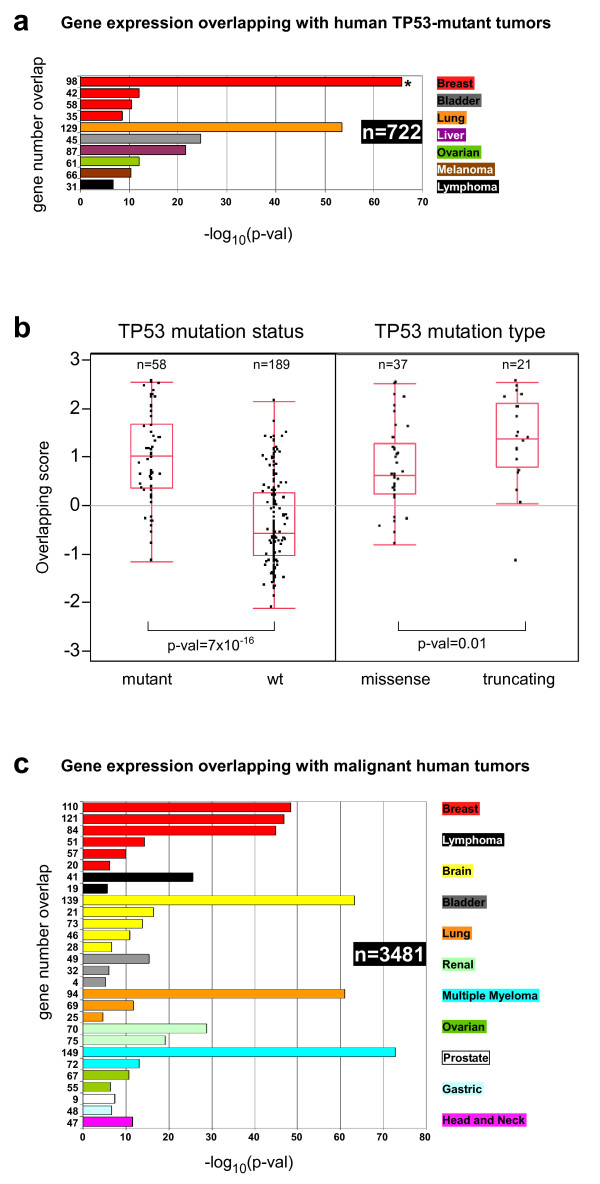
**Overlapping of mouse and human tumors**. (**a**) Gene expression overlapping between mouse tumors and human samples with TP53 mutations from 7 different cancer types is shown. n: total number of human tumors analyzed. Bar plots represent the significance of the overlap between overexpressed genes in tumors of p53-deficient mouse and genes overexpressed in human tumor samples with TP53 mutations (p-val, Fisher's exact test). Numbers at left represent the gene number overlap. Aside the plot is represented the color codes for each cancer tissue. Asterisk represents the most significant overlapping. (**b**) Mouse tumors as models for missense and truncating TP53 mutations. Gene expression values of the 98 genes overlapping between the mouse tumors and the breast cancer samples from Ivshina *et al. *[26] (asterisk in panel a) with TP53 mutations (overlapping score; mean = 0, stdv = 1) were plotted depending on the TP53 mutation status (left) or the mutation type (right). Number of tumor samples and significance of mean difference is shown. (**c**) Gene expression overlapping between mouse tumors and human samples with poor outcome from 11 different cancer types is shown. n: total number of human tumors analyzed. Bar plots represent the significance of the overlap between overexpressed genes in tumors of p53-deficient mouse and genes overexpressed in human tumor samples with poor outcome (p-val, Fisher's exact test). Numbers at left represent the gene number overlap. Aside the plot is represented the color codes for each cancer tissue.

To test whether the gene expression patterns that characterize Trp53^ΔEC ^and Rb^ΔEC^; Trp53^ΔEC ^mouse tumors (training dataset) are also present in human cancers with TP53 mutations and/or with poor clinical outcome, we performed an exhaustive comparison of the mouse tumor signature (682-probesets) with gene datasets of human cancer samples using the Oncomine human cancer genomics database (see Materials and Methods) [24,25]. First we analyzed the genes overexpressed in the mouse tumors (371 probesets), and compared them with the overexpressed genes in human samples bearing TP53 mutations. This meta-analysis showed a very significant overlap with many human epithelial and non-epithelial cancers, indicating that multiple genes overexpressed in the mouse epidermal tumors are in common with human tumors from distinct body sites and characterized by bearing mutant TP53 gene (Fig. 4a and Additional file 4). To study the existence of possible correlation with different types of p53 mutations in human tumors, we analyzed in further detail a breast cancer dataset containing gene expression and p53 mutation data of 247 patients [26], and which also showed the highest overlapping significance in the human p53-mutant tumors panel (asterisk in Fig. 4a) (98 overlapping genes, p-val = 1.6 × 10^-66^). As a measure of the status similarity with respect to mouse tumors, we calculated an overlapping score of each breast cancer sample (see Materials and Methods) and represented it as a function of TP53 mutational status. The mean differences and significance were calculated as the tumors were grouped by TP53 mutation status (mutant or wild type) or by mutation type (missense or truncating). As expected, the differences were highly significant between tumor samples bearing TP53 mutation or wild type (Fig. 4b, left panel), demonstrating that the mouse tumors expression profile could distinguish between both types of human tumors. Furthermore, the mean values were also significantly higher in the samples with truncating mutations when compared with missense mutations (Fig. 4b, right panel). Overall these analyses suggest that mouse tumors with somatic deficiency of both p53 alleles resemble human tumors with TP53 mutations, especially tumors that can produce truncated p53 proteins.

Next, we extracted common genes overexpressed in the mouse tumors and in human tumors bearing TP53 mutations. We found 51 that are overexpressed in 5 out of 10 studies of human tumors analyzed, representing a mouse and human tumor signature associated with TP53 mutation (51-gene signature) (Table 2).

**Table 2:** Mouse and human tumor signatures

Gene Symbol	Gene Title	Gene Function
Mouse and human tumor signature associated with TP53 mutation^1^: 51-gene signature		
ACTL6A	actin-like 6A	chromatin remodeling, histone H4 and H2A acetylation
ASF1B	ASF1 anti-silencing function 1 homolog B (S. cerevisiae)	chromatin assembly or disassembly, regulation of transcription
AURKA	aurora kinase A	mitotic cell cycle, spindle organization
BIRC5	baculoviral IAP repeat-containing 5	microtubule cytoskeleton organization, chromosome segregation, spindle checkpoint
BLM	Bloom syndrome, RecQ helicase-like	telomere maintenance, G2/M transition DNA damage checkpoint
BUB1	budding uninhibited by benzimidazoles 1 homolog (yeast)	mitotic cell cycle spindle assembly checkpoint
C21orf45	chromosome 21 open reading frame 45	mitosis
CCNA2	cyclin A2	mitotic cell cycle G2/M transition DNA damage checkpoint, response to estradiol and glucagon stimulus
CDC2	cell division cycle 2, G1 to S and G2 to M	APC-dependent proteasomal ubiquitin-dependent protein catabolic process
CDC20	cell division cycle 20 homolog (S. cerevisiae)	APC-dependent proteasomal ubiquitin-dependent protein catabolic process
CDC45L	CDC45 cell division cycle 45-like (S. cerevisiae)	DNA replication checkpoint, DNA replication initiation
CDC6	cell division cycle 6 homolog (S. cerevisiae)	DNA replication checkpoint, traversing start control point of mitotic cell cycle
CDC7	cell division cycle 7 homolog (S. cerevisiae)	G1/S transition of mitotic cell cycle, DNA replication
CDCA3	cell division cycle associated 3	mitosis
CDCA8	cell division cycle associated 8	mitotic metaphase, chromosome organization
CDKN3	cyclin-dependent kinase inhibitor 3	regulation of cyclin-dependent protein kinase activity, G1/S transition of mitotic cell cycle
CDT1	chromatin licensing and DNA replication factor 1	DNA replication checkpoint, regulation of DNA replication initiation
CENPA	centromere protein A	establishment of mitotic spindle orientation, nucleosome assembly
CENPE	centromere protein E, 312kDa	mitotic chromosome movement towards spindle pole, mitotic metaphase plate congression, kinetochore assembly
CEP55	centrosomal protein 55kDa	mitosis
CHEK1	CHK1 checkpoint homolog (S. pombe)	DNA damage checkpoint, G2/M transition of mitotic cell cycle
CKS2	CDC28 protein kinase regulatory subunit 2	regulation of cyclin-dependent protein kinase activity, spindle organization
CTPS	CTP synthase	CTP biosynthetic process, response to drug
DEPDC1	DEP domain containing 1	intracellular signaling cascade, GTPase activator activity
FEN1	flap structure-specific endonuclease 1	DNA repair, UV protection
FOXM1	forkhead box M1	regulation of transcription, DNA-dependent
GMPS	guanine monphosphate synthetase	GMP biosynthetic process
HMMR	hyaluronan-mediated motility receptor (RHAMM)	receptor activity, hyaluronic acid binding
KIF2C	kinesin family member 2C	establishment or maintenance of microtubule cytoskeleton polarity, regulation of chromosome segregation
KIF4A	kinesin family member 4A	microtubule-based movement, anterograde axon cargo transport
KIFC1	kinesin family member C1	mitotic sister chromatid segregation, microtubule-based movement
KPNA2	karyopherin alpha 2 (RAG cohort 1, importin alpha 1)	regulation of DNA recombination, M phase specific microtubule process, NLS-bearing substrate import into nucleus
MAD2L1	MAD2 mitotic arrest deficient-like 1 (yeast)	mitotic cell cycle spindle assembly checkpoint, APC-dependent proteasomal ubiquitin-dependent protein catabolic process
MCM2	minichromosome maintenance complex component 2	DNA replication initiation, regulation of transcription
MCM3	minichromosome maintenance complex component 3	DNA replication initiation, regulation of transcription
MCM4	minichromosome maintenance complex component 4	DNA replication initiation, regulation of transcription
MCM5	minichromosome maintenance complex component 5	DNA replication initiation, regulation of transcription
MCM7	minichromosome maintenance complex component 7	DNA replication initiation, regulation of transcription, response to DNA damage stimulus
MLF1IP	MLF1 interacting protein	regulation of transcription, DNA-dependent
NCAPH	non-SMC condensin I complex, subunit H	mitotic chromosome condensation
NDC80	NDC80 homolog, kinetochore complex component (S. cerevisiae)	establishment of mitotic spindle orientation, attachment of spindle microtubules to kinetochore
PLK1	polo-like kinase 1 (Drosophila)	mitotic prometaphase, positive regulation of ubiquitin-protein ligase activity during mitotic cell cycle
PLSCR1	phospholipid scramblase 1	phospholipid scrambling, platelet activation
PRC1	protein regulator of cytokinesis 1	mitotic spindle elongation, cytokinesis
RAD54L	RAD54-like (S. cerevisiae)	double-strand break repair via homologous recombination, response to ionizing radiation
TFDP2	Transcription factor Dp-2 (E2F dimerization partner 2)	regulation of transcription, DNA-dependent; cell cycle
TMEM48	transmembrane protein 48	protein and mRNA transport, nuclear pore complex assembly
TOP2A	Topoisomerase (DNA) II alpha 170kDa	DNA replication, topological change, ligation and repair, chromosome segregation, positive regulation of apoptosis
TPX2	TPX2, microtubule-associated, homolog (Xenopus laevis)	mitosis
TRIP13	thyroid hormone receptor interactor 13	double-strand break repair, transcription from RNA polymerase II promoter
UBE2C	ubiquitin-conjugating enzyme E2C	spindle organization, APC-dependent proteasomal ubiquitin-dependent protein catabolic process

Mouse and human tumor signature associated with poor prognosis^2^: 26-gene sinature		
AURKA*	aurora kinase A	mitotic cell cycle, spindle organization
AURKB	aurora kinase B	mitosis, protein localization to kinetochore
BIRC5*	baculoviral IAP repeat-containing 5	microtubule cytoskeleton organization, chromosome segregation, spindle checkpoint
BUB1*	budding uninhibited by benzimidazoles 1 homolog (yeast)	mitotic cell cycle spindle assembly checkpoint
BUB1B	budding uninhibited by benzimidazoles 1 homolog beta (yeast)	apoptosis, mitotic cell cycle checkpoint, APC-dependent proteasomal ubiquitin-dependent protein catabolic process
CCNA2*	cyclin A2	mitotic cell cycle G2/M transition DNA damage checkpoint, response to estradiol and glucagon stimulus
CDC2*	Cell division cycle 2, G1 to S and G2 to M	APC-dependent proteasomal ubiquitin-dependent protein catabolic process
CDC20*	cell division cycle 20 homolog (S. cerevisiae)	APC-dependent proteasomal ubiquitin-dependent protein catabolic process
CDKN3*	cyclin-dependent kinase inhibitor 3	regulation of cyclin-dependent protein kinase activity, G1/S transition of mitotic cell cycle
CENPA*	centromere protein A	establishment of mitotic spindle orientation, nucleosome assembly
CHEK1*	CHK1 checkpoint homolog (S. pombe)	DNA damage checkpoint, G2/M transition of mitotic cell cycle
CKS1B	CDC28 protein kinase regulatory subunit 1B	regulation of cyclin-dependent protein kinase activity
CKS2*	CDC28 protein kinase regulatory subunit 2	regulation of cyclin-dependent protein kinase activity, spindle organization
H2AFZ	H2A histone family, member Z	nucleosome assembly
HMMR*	hyaluronan-mediated motility receptor (RHAMM)	receptor activity, hyaluronic acid binding
KIF11	kinesin family member 11	mitotic centrosome separation, spindle pole body organization
KIF2C*	kinesin family member 2C	establishment or maintenance of microtubule cytoskeleton polarity, regulation of chromosome segregation
KPNA2*	karyopherin alpha 2 (RAG cohort 1, importin alpha 1)	regulation of DNA recombination, M phase specific microtubule process, NLS-bearing substrate import into nucleus
MAD2L1*	MAD2 mitotic arrest deficient-like 1 (yeast)	mitotic cell cycle spindle assembly checkpoint, APC-dependent proteasomal ubiquitin-dependent protein catabolic process
MCM4*	minichromosome maintenance complex component 4	DNA replication initiation, regulation of transcription
NCAPH*	non-SMC condensin I complex, subunit H	mitotic chromosome condensation
PLK1*	polo-like kinase 1 (Drosophila)	mitotic prometaphase, positive regulation of ubiquitin-protein ligase activity during mitotic cell cycle
PLK4	polo-like kinase 4 (Drosophila)	positive regulation of centriole replication
TOP2A*	Topoisomerase (DNA) II alpha 170kDa	DNA replication, topological change, ligation and repair, chromosome segregation, positive regulation of apoptosis
TPX2*	TPX2, microtubule-associated, homolog (Xenopus laevis)	mitosis
UBE2C*	ubiquitin-conjugating enzyme E2C	spindle organization, APC-dependent proteasomal ubiquitin-dependent protein catabolic process

^1^Genes were selected if they have a significant differential expression between wild type and TP53-mutated human tumors, in at list 5 out of 10 human studies (Fig. 4a)

^2^Genes were selected if they have a significant differential expression between primary tumors with poor versus good outcome in at list 14 out of 28 human studies (Fig. 4c)

*Genes common between both lists: 20-gene signature

On the other hand, we analyzed whether the mouse tumor gene signature can distinguish human aggressive tumor samples (tumors from which patients died at an early time post surgery or diagnosis) independently of the TP53 mutational status. Again, we found a highly significant overlap with human cancers of different origin (Fig. 4c, and Additional file 4), including carcinomas (breast, brain, bladder, and renal), and hematological cancers (lymphoma and myeloma). The overlapping of the tumor signature of p53-deficient mouse with genes differentially expressed in human aggressive tumors suggests that this signature could be predictive of malignant progression, in agreement with the observation that TP53 mutations have been associated with poor prognosis in human cancer [9,11]. To study this, we also extracted mouse tumor genes that display overexpression in highly metastatic human tumors (in 14 out of 28 studies). These genes (n = 26) represent a mouse and human tumor signature associated with poor prognosis (26-gene signature) (Table 2).

Remarkably, 20 genes (asterisks in Table 2) are common between the 26-gene signature (associated with poor prognosis) and the 51-gene signature (associated with TP53 mutation), corroborating, in agreement with others [9,11], that TP53-mutations or p53 functional inhibition is a common hallmark of human cancer malignancy. These 20 genes (20-gene signature) represent biomarkers of TP53 mutant and/or aggressive tumors, and, consequently, possible therapeutic targets.

It is worth mentioning that a similar analysis was also performed using genes showing reduced expression in mouse tumors as compared with normal mouse skin. However, the number of overlapping signatures and their significance is lower (Additional files 4 and 5).

### Validation of the 20-gene signature in the mouse testing dataset

**Figure 5:**
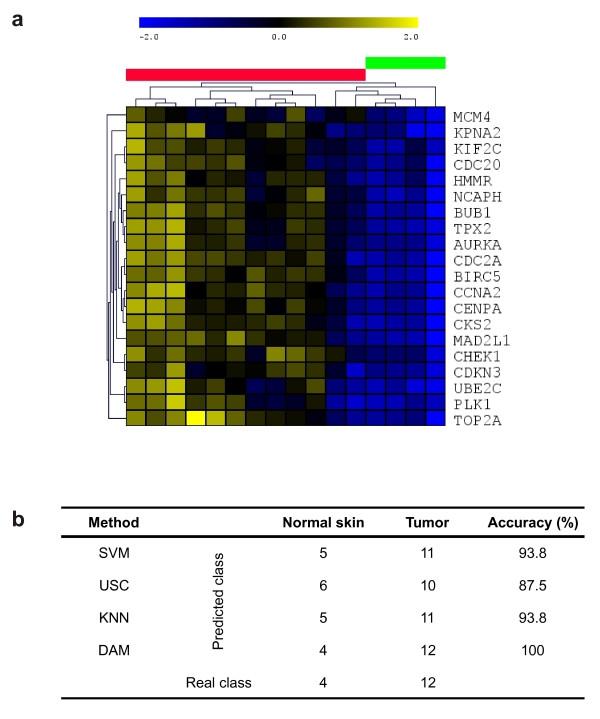
**Validation of 20-gene signature in the mouse testing dataset**. (**a**) Hierachical clustering (pearson distance, average linkage) of the 20-gene signature expression pattern in the testing dataset. Sample colors: green, normal control skin; red, mouse tumors. (**b**) Prediction analysis results. Shown is the real number of samples belonging to either normal skin class or tumor class, the classification result for each class using 4 different prediction methods, and the accuracy of the methods.

In order to validate the genes of the 20-gene signature as possible cancer targets or biomarkers, it is necessary to analyze its predictive capability to distinguish between normal skin and tumor samples arising in new mice. Gene expression values for the 20 genes from the testing mouse dataset were extracted (Fig. 5a), and the prediction accuracy to distinguish between normal or tumoral condition was calculated using Support Vector Machine (SVM) [27], Uncorrelated Shrunken Centroid Classification (USC) [28], K-Nearest Neighbor Classification (KNNC) [29] and Discriminant Analysis Module (DAM) [30]. The accuracy of the classifiers is very high, and varied between 87.5% (USC), 93.8% (SVM or KNNC), or 100% (DAM) (Fig. 5b). This result demonstrates that the 20-gene signature is a good predictor of carcinoma vs. normal skin samples, so the 20 genes could be considered good markers of Trp53^ΔEC ^and Rb^ΔEC^; Trp53^ΔEC ^mouse tumors and good targets for preclinical antitumor therapies.

### Patient stratification using 20-gene signature

**Figure 6:**
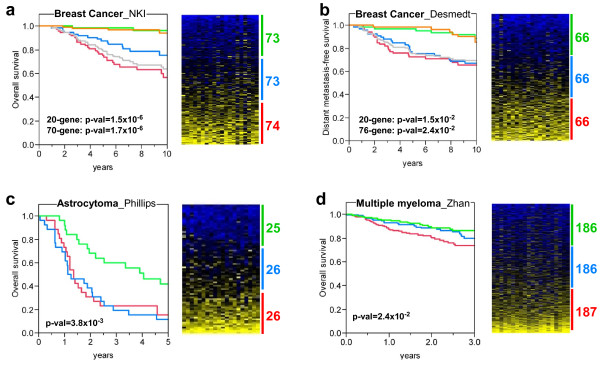
**20-gene signature can identify patient tumor samples with poor outcome from different human cancer types**. Three human cancer types were analyzed: breast cancer [31,32] (**a **and **b**), astrocytoma [51] (**c**) and multiple myeloma [50] (**d**). The probeset with maximal values was selected for those genes with multiple probesets. Expression values for each gene were mean centered (mean = 0, stdv = 1), and the sum of the expresion values of the 20 genes for each patient sample was computed (20-gene score, see Materials and Methods). Each dataset was divided in three groups of patients depending on the 20-gene score, and the survival of each group was represented in Kaplan-Meier curves. Note that the patients with lower scores (green color) display better survival than those with intermediate or higher scores (blue and red colors, respectively). Survival curves for good prognosis (orange line) and poor prognosis (grey line) breast cancer patients according to 70-gene and 76-gene prognosis profiles are showed (**a **and **b**, respectively). Significance of the survival differences is shown (p-val, Wilcoxon signed-rank test). On the right of each survival plot, there are heat maps of the corresponding datasets, and the numbers represent the number of patients in each 20-gene score group. From top to botton, the samples are ranked from lowest to highest 20-gene scores. From left to right, expression values of AURKA, BIRC5, BUB1, CCNA2, CDC2, CDC20, CDKN3, CENPA, CHEK1, CKS2, HMMR, KIF2C, KPNA2, MAD2L1, MCM4, NCAPH, PLK1, TOP2A, TPX2, and UBE2C.

To further confirm that the 20-gene signature obtained could be suitable for prediction analysis of human cancer outcome, we analyzed the overall survival of human patients depending on the expression pattern of this signature using four studies representing three different cancer types: breast cancer, astrocytoma, and multiple myeloma. To do this, we computed the sum of the expression values of the 20 genes (20-gene score) in each human tumor sample, we classified the samples depending on this score, and stratified the samples in three groups: low, intermediate and high score (Fig. 6). The association with survival of the resulting sample clusters was analyzed using Kaplan-Meier curves. The results showed that patients displaying low scores (this is, low expression values of the 20-gene signature) had a higher survival probability than those displaying intermediate or high scores (Fig. 6a-d), which suggests that the mouse-derived signature could help to determine the prognosis of human cancers.

Gene expression predictive tests have been previously developed for breast cancer with the aim to be implemented in clinical use. Thus, we wanted to analyze how the 20-gene derived patient stratification compares with prognostic signatures such as 70-gene [31] or 76-gene [32,33]. Survival curves according to 70-gene (Fig. 6a) and 76-gene (Fig. 6b) for good and poor prognosis breast cancer patients are very similar to the curves of low and high 20-gene scores, respectively. Furthermore, survival of patient groups using 20-gene scores displayed similar significance p-values compared to survival of 70-gene and 76-gene prognostic groups. Interestingly, 20-gene has 4 genes in common with 70-gene (BIRC5, BUB1, CENPA, and CKS2) and 2 genes in common with 76-gene (PLK1 and KPNA2).

**Figure 7:**
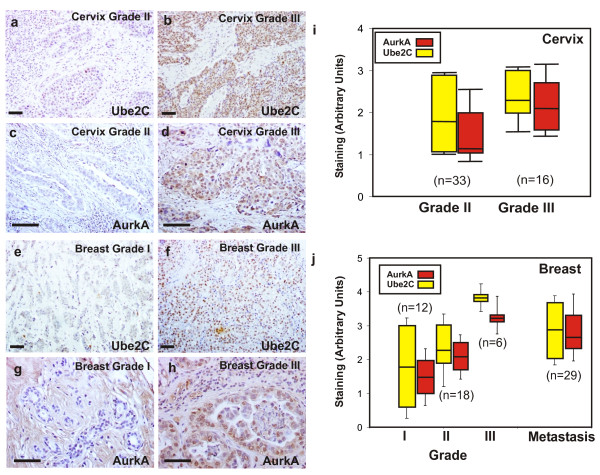
**UBE2C and AURKA expression in human breast and cervical carcinoma samples**. Representative examples of UBE2C (**a**, **b**, **e **and **f**) and AURKA (**c**, **d**, **g **and **h**) expression patterns in cervical (**a**, **b**, **c **and **d**) and breast carcinoma (**e**, **f**, **g **and **h**) primary tumors of different grades. Box plots of relative quantitation in primary cervical (**i**) and breast (**j**) tumors of different grades and breast metastases to lymph nodes (**j**) are represented.

Finally, we also investigated whether the genes identified could also behave as possible biomarkers for malignant progression of human cancer. To this, we analyzed the expression of two of the genes belonging to the 20-gene signature, UBE2C and AURKA, using immunohistochemistry on tissue array samples from human cervical (n = 55) and breast cancer (n = 86). The p53 pathway in both cancer types is frequently inhibited, either by expression of human papillomavirus E6 oncogene, which induces p53 protein degradation in cervical carcinomas, or by mutation of the TP53 gene in breast carcinomas. As the tissue collections include primary cancer samples of different tumor grades, we could relate the expression of both proteins with tumor grade. Furthermore, in the case of breast cancer, we could assess the expression in metastases. The analysis of immunohistochemical data (Fig. 7) revealed that UBE2C and AURKA expression levels are higher in undifferentiated tumors of cervical (p < 0.0001 and p < 0.004, for UBE2C and AURKA respectively) and breast (p < 0.001 and p < 0.0001, for UBE2C and AURKA respectively) cancer samples. Moreover, in both cases there is significant correlation between the expression levels of these putative biomarkers and grade (p < 0.001 and p < 0.02, for cervix and breast tumors respectively). As undifferentiated tumors are more aggressive and metastatic, we can suggest that UBE2C and AURKA are overexpressed in malignant tumors. Furthermore, expression levels are higher in metastases of breast cancer tumors (p < 0.001 and p < 0.01, for UBE2C and AURKA respectively), which again points to a role for these proteins in metastatic behavior.

Collectively, these results demonstrate that the mouse tumor derived 20-gene signature efficiently distinguishes groups of patients with different outcomes and from different types of human cancer.

## Discussion

Mouse models of human cancer could potentially be used as tools for preclinical analysis of antitumor therapies. However, before doing so, a full molecular characterization of the animal tumors is necessary to be able to compare them with the human tumors counterpart and to validate them. Gene expression studies are perfectly suited approaches for such validation and comparison. Moreover, interspecies comparison could also be useful to determine which genes display similar expression patterns in human and mouse models so they can be considered targets for therapy and/or cancer biomarkers in preclinical settings. Here we report the gene expression profiling of mouse tumors arising in epidermis as a consequence of the somatic ablation of either *Trp53 *or *Trp53 *and *Rb *tumor suppressor genes.

The supervised gene expression profiles of the mouse tumors obtained demonstrated that there are no major differences between the two genotypes. This finding is in agreement with our previous results using different allelic combinations and indicating that loss of Rb mediated acceleration of tumor appearance but did not affect the histological grade growth or aggressiveness of overt tumors [15]. The fact that most of the overexpressed genes are involved in cell cycle or mitotic control as well as chromosome instability (see Fig. 3c) is also in agreement with our previous data indicating that development of tumors in the Trp53^ΔEC ^mice is associated with early chromosome aberrations due to deregulated centrosome division occurring in pretumoral epidermis [23]. Under this context, the increase in proliferation due to Rb loss [17] can accelerate the process of tumorigenesis by increasing the number of cells subject to such chromosome alterations.

Our data also suggested another important hypothesis: since mutations in TP53 and chromosome instability are associated with increased aggressiveness and malignancy in human patients, we can speculate that mouse p53-deficient epidermal tumors can represent a well suited model for human malignant cancer analysis. Three major findings of the mouse-to-human microarray gene expression comparison presented here support such hypothesis: i) there is a significant overlapping of gene expression patterns between mouse tumors and human cancers bearing mutant TP53; ii) the gene expression signatures typical of human ES cells are displayed by mouse tumors; and iii) the overlapping of overexpressed genes between mouse tumors and highly malignant human primary tumors from various origins.

The similarities that we have found between mouse tumors and human cancer samples from different tissues and origin could be explained by the undifferentiated status of animal tumor samples. In this sense, the 20-gene signature (asterisks in Table 2) shares 13 genes with a meta-signature of undifferentiated human cancer (69 genes) previously published [34], demonstrating the molecular similarities between mouse tumors and human high grade samples. Furthermore, the undifferentiated and aggressive features of the mouse tumors could be related to the expression pattern representative of an ES cell-like phenotype. GSEA using signatures of human ES cell targets and key regulators of ES cell identity (Oct4, Sox2, and Nanog) or Myc oncogene demonstrates that mouse tumors display similar expression pattern of ES cells.

One important feature of the spontaneous tumors arising in both genotypes is the rapid loss of the differentiated phenotype. The microarray analysis corroborates the changes observed by histology studies and confirm, at the molecular level, that an important fraction of the tumors undergo EMT. Since EMT also correlates with metastatic properties, this result would also support a highly metastatic behavior of the mouse epidermal tumors. Analyses to characterize these features in more detail are underway.

These observations also reinforce the possibility that mouse models can be useful as tools for preclinical analysis of potential antitumor therapies targeted against specific signaling pathways or gene products. By means of meta-analysis approaches and interspecies comparison we developed a signature composed by 20 genes, which displays the following attributes: i) it is composed by genes displaying increased expression in tumors compared to normal tissue, being possible targets with important functions in the carcinoma maintenance/aggressiveness; ii) it is independent of the genotype (Trp53^ΔEC ^or Rb^ΔEC^; Trp53^ΔEC^) or the histological subtype; and more importantly, iii) it can identify human primary tumors bearing TP53 mutations and/or displaying a more aggressive malignant behavior. In consequence, we postulate that these 20 genes, besides of being considered biomarkers of malignant human cancer progression, could also be useful cancer therapeutic targets, whose inhibition can be preclinically tested in our mouse models.

Consistent with the functional roles of p53, the 20-gene signature contain genes involved in DNA replication and repair, or genomic instability and cell cycle checkpoint (Table 2). This finding is coincident with previous reports showing that TP53 mutations are associated with increased global genomic instability [22], and with a report describing a signature of chromosomal instability (CIN25) inferred from gene expression profiles that predicts clinical outcome in multiple human cancers [35]. Thus, the results suggest that chromosomal instability mediated by the loss of TP53 could be the driving force of metastatic behavior in primary tumors with TP53 mutations.

Epidemiological studies of human cancer demonstrate that somatic mutations in the TP53 gene are mostly missense (73.6% of all mutations). Some missense mutations produce p53-mutant proteins that have been associated with gain of function, or dominant-negative activities. Supported by studies in genetically engineered mice [13], gain of function activity renders a more transformation prone phenotype and confers major metastatic advantages. However, it is important to note that such features have been characterized in mice when the mutations were introduced in combination with specific oncogenes such as K-ras [36] or in the absence of MDM2 [37]. In addition, dominant negative of mutated p53 is only apparent upon external carcinogenic challenges [38]. On the other hand, an important proportion of TP53 mutations found in human cancers would give rise to truncated p53 proteins, such as nonsense, frameshift and large deletion mutations (16.6% of all mutations). In this sense, our animal models of somatic inactivation of the Trp53 mouse gene could represent a suitable model for these types of human mutations. Analysis of the expression deregulation similarities of the mouse tumors and human samples with either truncating or missense TP53 mutations (Fig. 4b) suggest that animal samples share molecular features with both types of mutations, but more significantly with those producing truncated p53 protein. Recently, it has been described that an important proportion of BRCA1-related human breast tumors display TP53 mutations that produce truncated p53 proteins [39]. Approximately half of all hereditary breast cancers are due to loss of BRCA1 or BRCA2 function. Thus, cancer treatments that can restore TP53 pathway function in the mouse models could then be used to treat these BRCA1-related breast tumors, where the p53 loss has been suggested to be essential for tumorigenesis.

The results shown here constitute a comprehensive metagenomic comparison of mouse p53-deficient skin SCCs with human cancer in which we describe common genes of p53-dependent malignancy. These genes are markers of malignant cancer, and potential targets for antitumor therapy. Furthermore, as these genes share expression patterns in both species and in different types of human cancer, our mouse models constitute validated models to initiate preclinical analysis of antitumor therapies that could be useful against p53-mutated human cancer.

## Methods

### Microarray analysis of mouse skin tumors

RNA was obtained from 9 normal wild type control skin samples and 27 tumors of both genotypes, and purified from mice tissue as previously described [15]. Hybridization was done to Affymetrix Mouse Gene expression MOE430 2.0 array. Raw and processed data for training and testing mouse datasets were deposited in the GEO database with the accession identifiers GSE11990 and GSE19616, respectively. Supervised analysis of differential expression between tumors with different histological grade or arising in the two different genotypes was done using Ttest available in the open source software Multiexperiment Viewer 4.5 (MeV 4.5) [40]. The p-values were corrected using FDR. To obtain a tumor signature of p53-deficient mice, differential expression of mouse tumors compared to normal skin tissue was performed using Ttest and Significant Analysis of Microarrays (SAM) [41]. Probes were selected if they passed two criteria: i) Ttest analysis with FDR corrected p-val < 3 × 10^-7^; and ii) SAM analysis with q-val < 1 × 10^-3 ^for FDR. A number of 682 probesets were selected as differentially regulated, being 371 overexpressed and 311 underexpressed in mouse tumors (Fig. 3, Additional files 2 and 3). MOE430 2.0 Affymetrix chip probeset IDs were mapped to human using Ailun web utility [42].

### Enrichment analysis of Gene Ontology terms

Probesets identifiers of differentially expressed genes were uploaded into DAVID Functional Annotation web tool, which computes enrichment of Gene Ontology biological processes terms using EASE score [43,44].

### Enrichment analysis in human stem cells signatures

GSEA [20,21] was used to analyze the enrichment of human embryonic stem cell gene signatures within the mouse tumors when compared to normal skin. Gene sets were downloaded from Ben-Porath *et al. *[19], and fall into four groups: (i) ES expressed genes: two sets of genes overexpressed in ES cells compared to other cells and tissues according to a multistudy compilation and meta-analysis [45]; (ii) Nanog, Oct4 and Sox2 (NOS) targets: four sets of genes whose promoters are bound and activated in human ES cells by each of these regulators of ES cell identity, or co-activated by all three [46], and an additional set (NOS TFs) including a subset of NOS activation targets encoding transcription regulators; (iii) Polycomb targets: four sets representing genes bound by the Polycomb repressive complex 2 (PRC2) in human ES cells [47]; and (iv) Myc targets: two sets of genes bound and activated by c-Myc, identified in two independent studies [48,49].

### Overlapping analysis in human cancer gene expression studies

We used Oncomine Gene Expression Signatures database to search for overlapping [24]. Association of the mapped signatures with the database signatures was tested using Fisher's exact test, and was considered significant for Odds Ratio >1.25, and p-val < 0.01. Genes overexpressed or underexpressed in the tumor signature of p53-deficient mouse were mapped to human gene symbols and loaded into the Oncomine database. Although both sets of genes display similar trends, the significance was lower for those underexpressed. We have searched for overlaps using different filtering criteria, based on the type of human cancer comparison performed. These criteria were: "Molecular Subtype: Mutation" and "Clinical Outcome". In the case of the overlapping with the Ivshina *et al. *study of TP53 mutational status in breast cancer samples [26] (asterisk in Fig. 4a), as a measure of status similarity with respect to mouse tumors, we calculated the sum of the expression values of the 98 common genes in each breast cancer sample. The higher the sum, the greater the overlap with the mouse tumor signature.

### Validation analysis in the mouse tumor testing dataset

For both the training (GSE11990) and testing mouse datasets (GSE19616) the gene expression values for the 20-gene signature were extracted. The probeset with maximal value was selected for genes with more than one. Similar results were obtained using the median value of probesets. For each method, the classifier was trained in the GSE11990 dataset and tested in the GSE19616 dataset. Prediction methods (SVM, USC, KNNC and DAM) were calculated using default settings within the MeV 4.5 software.

### Immunohistochemistry on human tissue arrays

FFPE tissue arrays of breast and cervical carcinomas were purchased to Cybrdi, Inc. (Maryland, USA). Individual clinical specimens were pathologically confirmed. Immunohistochemistry was done using standard protocols on deparaffinized sections using a polyclonal rabbit antibody to human UBE2C or Aurora kinase A (AbCam). The slides were microwaved for 15 min. Biotin-conjugated secondary antibody was purchased from Jackson Immuno-Research Laboratories and used at 1:1,000. Peroxidase was visualized using avidin-biotin complex method and 3,3-diaminobenzidine kit (Vector). Double-blind analysis was performed to assign a staining score (0 to 5) for each sample considering the intensity and the percentage of tumor cells stained. Statistical analyses (χ^2 ^and Cox correlation) were performed using SPSS software.

### External microarray datasets of human cancer

Gene expression and clinical information of human cancer survival curves were downloaded either from GEO website (multiple myeloma_Zhan dataset (GSE2658) [50], astrocytoma_Phillips dataset (GSE4271) [51], and breast cancer_Desmedt (GSE7390) [32]), or from Rosetta Inpharmatics website (breast cancer_NKI [31]).

### Ethics Statement

All animals were handled in strict accordance with good animal practice as defined by the relevant international laboratory animal welfare bodies (FELASA), and all animal work was approved by the Animal Ethical Committee and conducted in compliance with Centro de Investigaciones Energéticas, Medioambientales y Tecnológicas (CIEMAT) Guidelines.

## Competing interests

The authors declare that they have no competing interests.

## Authors' contributions

All authors have read and approved the final manuscript. ABM-C, MS, CS, CL, CSL, and CC contributed to animal propagation; ABM-C and MS collected mouse skin samples; ABM-C, MD and AB-P performed RNA purification of animal samples; GG and MD performed immunohistochemistry analyses. RG-E performed microarray analysis and wrote the manuscript; and RG-E and J-MP planned and supervised the project.

## Supplementary Material

Additional file 1 **Differential expression analysis of tumors arising in either Trp53^ΔEC ^or Rb^ΔEC^; Trp53^ΔEC ^models from GSE11990 and GSE19616 datasets**. The figure represent hierarchical clustering of genes selected using Ttest with corrected p-val (FDR < 0.1). Click here for file

Additional file 2 **371 probesets overexpressed in mouse p53-tumors**. The table includes the probesets IDs, fold change and gene symbol corresponding to the overexpressed Affymetrix probesets in the mouse tumors of the training dataset. Click here for file

Additional file 3 **311 probesets underexpressed in mouse p53-tumors**. The table includes the probesets IDs, fold change and gene symbol corresponding to the underexpressed Affymetrix probesets in the mouse tumors of the training dataset. Click here for file

Additional file 4 **Significant overlaps of human-mapped overexpressed or underexpressed genes from tumors of p53-deficient mouse with human tumors using "Molecular Subtype Mutations" and "Clinical Outcome" analyses**. The file includes a table with the Concept Name, gene number overlap and overlapping significance of the signature of p53-deficient mouse compared to human signatures with p53-mutations or with poor outcome, and also includes the bibliographic references of the human signatures. Click here for file

Additional file 5 **Overlapping between underexpressed genes in the tumor signature of p53-deficient mouse and genes underexpressed in human primary tumors with malignant behavior**. The figure represents the overlapping significance with signatures of poor-outcome human tumors from different anatomical locations (A) and the list of genes which are underexpressed in the majority of the human signatures (B). Click here for file

## References

[B1] Ellwood-Yen K Graeber TG Wongvipat J Iruela-Arispe ML Zhang J Matusik R Thomas GV Sawyers CL Myc-driven murine prostate cancer shares molecular features with human prostate tumors Cancer Cell 2003 4 223 238 10.1016/S1535-6108(03)00197-1 14522256

[B2] Sweet-Cordero A Mukherjee S Subramanian A You H Roix JJ Ladd-Acosta C Mesirov J Golub TR Jacks T An oncogenic KRAS2 expression signature identified by cross-species gene-expression analysis Nat Genet 2005 37 48 55 15608639 10.1038/ng1490

[B3] Hodgson G Hager JH Volik S Hariono S Wernick M Moore D Nowak N Albertson DG Pinkel D Collins C Genome scanning with array CGH delineates regional alterations in mouse islet carcinomas Nat Genet 2001 29 459 464 10.1038/ng771 11694878

[B4] Maser RS Choudhury B Campbell PJ Feng B Wong KK Protopopov A O'Neil J Gutierrez A Ivanova E Perna I Chromosomally unstable mouse tumours have genomic alterations similar to diverse human cancers Nature 2007 447 966 971 10.1038/nature05886 17515920 PMC2714968

[B5] O'Hagan RC Chang S Maser RS Mohan R Artandi SE Chin L DePinho RA Telomere dysfunction provokes regional amplification and deletion in cancer genomes Cancer Cell 2002 2 149 155 10.1016/S1535-6108(02)00094-6 12204535

[B6] Zender L Spector MS Xue W Flemming P Cordon-Cardo C Silke J Fan ST Luk JM Wigler M Hannon GJ Identification and validation of oncogenes in liver cancer using an integrative oncogenomic approach Cell 2006 125 1253 1267 10.1016/j.cell.2006.05.030 16814713 PMC3026384

[B7] Menendez D Inga A Resnick MA The expanding universe of p53 targets Nat Rev Cancer 2009 9 724 737 10.1038/nrc2730 19776742

[B8] Vousden KH Prives C Blinded by the Light: The Growing Complexity of p53 Cell 2009 137 413 431 10.1016/j.cell.2009.04.037 19410540

[B9] Petitjean A Mathe E Kato S Ishioka C Tavtigian SV Hainaut P Olivier M Impact of mutant p53 functional properties on TP53 mutation patterns and tumor phenotype: lessons from recent developments in the IARC TP53 database Hum Mutat 2007 28 622 629 10.1002/humu.20495 17311302

[B10] Vogelstein B Lane D Levine AJ Surfing the p53 network Nature 2000 408 307 310 10.1038/35042675 11099028

[B11] Toledo F Wahl GM Regulating the p53 pathway: in vitro hypotheses, in vivo veritas Nat Rev Cancer 2006 6 909 923 10.1038/nrc2012 17128209

[B12] Brosh R Rotter V When mutants gain new powers: news from the mutant p53 field Nat Rev Cancer 2009 9 701 713 19693097 10.1038/nrc2693

[B13] Donehower LA Lozano G 20 years studying p53 functions in genetically engineered mice Nat Rev Cancer 2009 9 831 841 19776746 10.1038/nrc2731

[B14] Jonkers J Meuwissen R van der Gulden H Peterse H van der Valk M Berns A Synergistic tumor suppressor activity of BRCA2 and p53 in a conditional mouse model for breast cancer Nat Genet 2001 29 418 425 10.1038/ng747 11694875

[B15] Martinez-Cruz AB Santos M Lara MF Segrelles C Ruiz S Moral M Lorz C Garcia-Escudero R Paramio JM Spontaneous squamous cell carcinoma induced by the somatic inactivation of retinoblastoma and Trp53 tumor suppressors Cancer Res 2008 68 683 692 10.1158/0008-5472.CAN-07-3049 18245467

[B16] Peinado H Olmeda D Cano A Snail, Zeb and bHLH factors in tumour progression: an alliance against the epithelial phenotype? Nat Rev Cancer 2007 7 415 428 10.1038/nrc2131 17508028

[B17] Ruiz S Santos M Segrelles C Leis H Jorcano JL Berns A Paramio JM Vooijs M Unique and overlapping functions of pRb and p107 in the control of proliferation and differentiation in epidermis Development 2004 131 2737 2748 10.1242/dev.01148 15148303

[B18] Ruiz S Santos M Lara MF Segrelles C Ballestin C Paramio JM Unexpected roles for pRb in mouse skin carcinogenesis Cancer Res 2005 65 9678 9686 10.1158/0008-5472.CAN-05-1853 16266987

[B19] Ben-Porath I Thomson MW Carey VJ Ge R Bell GW Regev A Weinberg RA An embryonic stem cell-like gene expression signature in poorly differentiated aggressive human tumors Nat Genet 2008 40 499 507 10.1038/ng.127 18443585 PMC2912221

[B20] Mootha VK Lindgren CM Eriksson KF Subramanian A Sihag S Lehar J Puigserver P Carlsson E Ridderstrale M Laurila E PGC-1alpha-responsive genes involved in oxidative phosphorylation are coordinately downregulated in human diabetes Nat Genet 2003 34 267 273 10.1038/ng1180 12808457

[B21] Subramanian A Tamayo P Mootha VK Mukherjee S Ebert BL Gillette MA Paulovich A Pomeroy SL Golub TR Lander ES Mesirov JP Gene set enrichment analysis: a knowledge-based approach for interpreting genome-wide expression profiles Proc Natl Acad Sci USA 2005 102 15545 15550 10.1073/pnas.0506580102 16199517 PMC1239896

[B22] Eyfjord JE Thorlacius S Steinarsdottir M Valgardsdottir R Ogmundsdottir HM Anamthawat-Jonsson K p53 abnormalities and genomic instability in primary human breast carcinomas Cancer Res 1995 55 646 651 7530599

[B23] Martinez-Cruz AB Santos M Garcia-Escudero R Moral M Segrelles C Lorz C Saiz C Buitrago-Perez A Costa C Paramio JM Spontaneous tumor formation in Trp53-deficient epidermis mediated by chromosomal instability and inflammation Anticancer Res 2009 29 3035 3042 19661312

[B24] Rhodes DR Kalyana-Sundaram S Tomlins SA Mahavisno V Kasper N Varambally R Barrette TR Ghosh D Varambally S Chinnaiyan AM Molecular concepts analysis links tumors, pathways, mechanisms, and drugs Neoplasia 2007 9 443 454 10.1593/neo.07292 17534450 PMC1877973

[B25] Rhodes DR Kalyana-Sundaram S Mahavisno V Varambally R Yu J Briggs BB Barrette TR Anstet MJ Kincead-Beal C Kulkarni P Oncomine 3.0: genes, pathways, and networks in a collection of 18,000 cancer gene expression profiles Neoplasia 2007 9 166 180 10.1593/neo.07112 17356713 PMC1813932

[B26] Ivshina AV George J Senko O Mow B Putti TC Smeds J Lindahl T Pawitan Y Hall P Nordgren H Genetic reclassification of histologic grade delineates new clinical subtypes of breast cancer Cancer Res 2006 66 10292 10301 10.1158/0008-5472.CAN-05-4414 17079448

[B27] Brown MP Grundy WN Lin D Cristianini N Sugnet CW Furey TS Ares M Jr Haussler D Knowledge-based analysis of microarray gene expression data by using support vector machines Proc Natl Acad Sci USA 2000 97 262 267 10.1073/pnas.97.1.262 10618406 PMC26651

[B28] Yeung KY Bumgarner RE Multiclass classification of microarray data with repeated measurements: application to cancer Genome Biol 2003 4 R83 10.1186/gb-2003-4-12-r83 14659020 PMC329422

[B29] Theilhaber J Connolly T Roman-Roman S Bushnell S Jackson A Call K Garcia T Baron R Finding genes in the C2C12 osteogenic pathway by k-nearest-neighbor classification of expression data Genome Res 2002 12 165 176 10.1101/gr.182601 11779842 PMC155256

[B30] Nguyen DV Rocke DM Multi-class cancer classification via partial least squares with gene expression profiles Bioinformatics 2002 18 1216 1226 10.1093/bioinformatics/18.9.1216 12217913

[B31] van de Vijver MJ He YD van't Veer LJ Dai H Hart AA Voskuil DW Schreiber GJ Peterse JL Roberts C Marton MJ A gene-expression signature as a predictor of survival in breast cancer N Engl J Med 2002 347 1999 2009 10.1056/NEJMoa021967 12490681

[B32] Desmedt C Piette F Loi S Wang Y Lallemand F Haibe-Kains B Viale G Delorenzi M Zhang Y d'Assignies MS Strong time dependence of the 76-gene prognostic signature for node-negative breast cancer patients in the TRANSBIG multicenter independent validation series Clin Cancer Res 2007 13 3207 3214 10.1158/1078-0432.CCR-06-2765 17545524

[B33] Wang Y Klijn JG Zhang Y Sieuwerts AM Look MP Yang F Talantov D Timmermans M Meijer-van Gelder ME Yu J Gene-expression profiles to predict distant metastasis of lymph-node-negative primary breast cancer Lancet 2005 365 671 679 15721472 10.1016/S0140-6736(05)17947-1

[B34] Rhodes DR Yu J Shanker K Deshpande N Varambally R Ghosh D Barrette T Pandey A Chinnaiyan AM Large-scale meta-analysis of cancer microarray data identifies common transcriptional profiles of neoplastic transformation and progression Proc Natl Acad Sci USA 2004 101 9309 9314 10.1073/pnas.0401994101 15184677 PMC438973

[B35] Carter SL Eklund AC Kohane IS Harris LN Szallasi Z A signature of chromosomal instability inferred from gene expression profiles predicts clinical outcome in multiple human cancers Nat Genet 2006 38 1043 1048 10.1038/ng1861 16921376

[B36] Caulin C Nguyen T Lang GA Goepfert TM Brinkley BR Cai WW Lozano G Roop DR An inducible mouse model for skin cancer reveals distinct roles for gain- and loss-of-function p53 mutations J Clin Invest 2007 117 1893 1901 10.1172/JCI31721 17607363 PMC1904325

[B37] Terzian T Suh YA Iwakuma T Post SM Neumann M Lang GA Van Pelt CS Lozano G The inherent instability of mutant p53 is alleviated by Mdm2 or p16INK4a loss Genes Dev 2008 22 1337 1344 10.1101/gad.1662908 18483220 PMC2377188

[B38] Wijnhoven SW Speksnijder EN Liu X Zwart E vanOostrom CT Beems RB Hoogervorst EM Schaap MM Attardi LD Jacks T Dominant-negative but not gain-of-function effects of a p53.R270H mutation in mouse epithelium tissue after DNA damage Cancer Res 2007 67 4648 4656 10.1158/0008-5472.CAN-06-4681 17510390

[B39] Holstege H Joosse SA van Oostrom CT Nederlof PM de Vries A Jonkers J High incidence of protein-truncating TP53 mutations in BRCA1-related breast cancer Cancer Res 2009 69 3625 3633 10.1158/0008-5472.CAN-08-3426 19336573

[B40] Saeed AI Sharov V White J Li J Liang W Bhagabati N Braisted J Klapa M Currier T Thiagarajan M TM4: a free, open-source system for microarray data management and analysis Biotechniques 2003 34 374 378 12613259 10.2144/03342mt01

[B41] Tusher VG Tibshirani R Chu G Significance analysis of microarrays applied to the ionizing radiation response Proc Natl Acad Sci USA 2001 98 5116 5121 10.1073/pnas.091062498 11309499 PMC33173

[B42] Chen R Li L Butte AJ AILUN: reannotating gene expression data automatically Nat Methods 2007 4 879 10.1038/nmeth1107-879 17971777 PMC2716375

[B43] Dennis G Jr Sherman BT Hosack DA Yang J Gao W Lane HC Lempicki RA DAVID: Database for Annotation, Visualization, and Integrated Discovery Genome Biol 2003 4 P3 10.1186/gb-2003-4-5-p3 12734009 PMC193660

[B44] Hosack DA Dennis G Jr Sherman BT Lane HC Lempicki RA Identifying biological themes within lists of genes with EASE Genome Biol 2003 4 R70 10.1186/gb-2003-4-10-r70 14519205 PMC328459

[B45] Assou S Le Carrour T Tondeur S Strom S Gabelle A Marty S Nadal L Pantesco V Reme T Hugnot JP A meta-analysis of human embryonic stem cells transcriptome integrated into a web-based expression atlas Stem Cells 2007 25 961 973 10.1634/stemcells.2006-0352 17204602 PMC1906587

[B46] Boyer LA Lee TI Cole MF Johnstone SE Levine SS Zucker JP Guenther MG Kumar RM Murray HL Jenner RG Core transcriptional regulatory circuitry in human embryonic stem cells Cell 2005 122 947 956 10.1016/j.cell.2005.08.020 16153702 PMC3006442

[B47] Lee TI Jenner RG Boyer LA Guenther MG Levine SS Kumar RM Chevalier B Johnstone SE Cole MF Isono K Control of developmental regulators by Polycomb in human embryonic stem cells Cell 2006 125 301 313 10.1016/j.cell.2006.02.043 16630818 PMC3773330

[B48] Fernandez PC Frank SR Wang L Schroeder M Liu S Greene J Cocito A Amati B Genomic targets of the human c-Myc protein Genes Dev 2003 17 1115 1129 10.1101/gad.1067003 12695333 PMC196049

[B49] Li Z Van Calcar S Qu C Cavenee WK Zhang MQ Ren B A global transcriptional regulatory role for c-Myc in Burkitt's lymphoma cells Proc Natl Acad Sci USA 2003 100 8164 8169 10.1073/pnas.1332764100 12808131 PMC166200

[B50] Zhan F Huang Y Colla S Stewart JP Hanamura I Gupta S Epstein J Yaccoby S Sawyer J Burington B The molecular classification of multiple myeloma Blood 2006 108 2020 2028 10.1182/blood-2005-11-013458 16728703 PMC1895543

[B51] Phillips HS Kharbanda S Chen R Forrest WF Soriano RH Wu TD Misra A Nigro JM Colman H Soroceanu L Molecular subclasses of high-grade glioma predict prognosis, delineate a pattern of disease progression, and resemble stages in neurogenesis Cancer Cell 2006 9 157 173 10.1016/j.ccr.2006.02.019 16530701

