# Supplementary material for: Gene expression profiling of mouse p53-deficient epidermal carcinoma defines molecular determinants of human cancer malignancy
Source: Mol Cancer. 2010 Jul 14;9:193. doi: 10.1186/1476-4598-9-193 (PMC2913987; doi:10.1186/1476-4598-9-193)

**Additional file 1.** Differential expression analysis of tumors arising in either Trp53^ΔEC^ or Rb^ΔEC^; Trp53^ΔEC^ models from GSE11990 and GSE19616 datasets. Hierarchical clustering of genes selected using Ttest with corrected p-val (FDR<0.1) is shown. Sample colors: orange, tumors from Trp53^ΔEC^ mice; red, tumors from Rb^ΔEC^; Trp53^ΔEC^ mice. NA: unknown gene symbol.


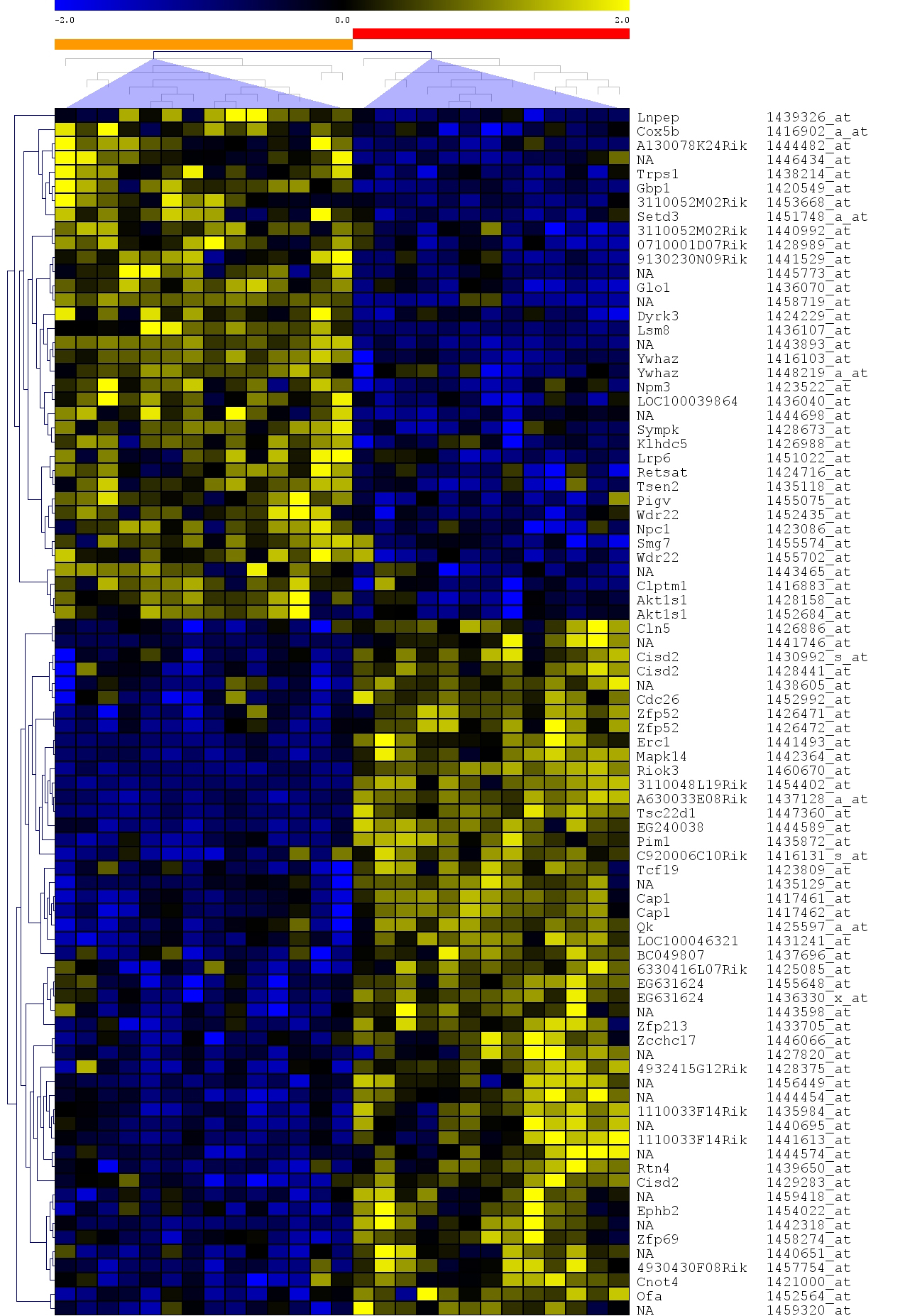

Supplement: Additional file 1 — Differential expression analysis of tumors arising in either Trp53ΔEC or RbΔEC; Trp53ΔEC models from GSE11990 and GSE19616 datasets. The figure represent hierarchical clustering of genes selected using Ttest with corrected p-val (FDR < 0.1). [file 1476-4598-9-193-S1.DOC]
