# Supplementary material for: Gene expression profiling of mouse p53-deficient epidermal carcinoma defines molecular determinants of human cancer malignancy
Source: Mol Cancer. 2010 Jul 14;9:193. doi: 10.1186/1476-4598-9-193 (PMC2913987; doi:10.1186/1476-4598-9-193)
Supplement: Additional file 2 — 371 probesets overexpressed in mouse p53-tumors. The table includes the probesets IDs, fold change and gene symbol corresponding to the overexpressed Affymetrix probesets in the mouse tumors of the training dataset. [file 1476-4598-9-193-S2.DOC]

**Additional file 2.** 371 probesets overexpressed in mouse p53-tumors.

| **Probesets ID** | **Fold Change** | **Gene Symbol** |
| --- | --- | --- |
| 1449254_at | 62.31 | Spp1 |
| 1424046_at | 19.70 | Bub1 |
| 1417541_at | 16.23 | Hells |
| 1443621_at | 16.22 | Fbxo39 |
| 1445226_at | 15.94 | BC023969 |
| 1424292_at | 14.88 | Depdc1a |
| 1429295_s_at | 14.63 | Trip13 |
| 1429734_at | 14.63 | 4632434I11Rik |
| 1452242_at | 13.76 | Cep55 |
| 1449578_at | 13.62 | Supt16h |
| 1416757_at | 12.86 | Zwilch |
| 1424454_at | 12.53 | Tmem87a |
| 1438403_s_at | 12.30 | --- |
| 1448754_at | 12.23 | LOC100045055 /// Rbp1 |
| 1438824_at | 12.11 | Slc20a1 |
| 1418569_at | 12.04 | Fblim1 |
| 1450886_at | 12.03 | Gsg2 |
| 1449699_s_at | 11.46 | C330027C09Rik |
| 1452458_s_at | 11.21 | Ppil5 |
| 1450677_at | 11.15 | Chek1 |
| 1417019_a_at | 11.14 | Cdc6 |
| 1429171_a_at | 10.91 | Ncapg |
| 1419513_a_at | 10.76 | Ect2 |
| 1426165_a_at | 10.57 | Casp3 |
| 1439040_at | 10.29 | Cenpe |
| 1424278_a_at | 10.08 | Birc5 |
| 1424511_at | 9.61 | Aurka |
| 1450156_a_at | 9.36 | Hmmr |
| 1425908_at | 9.10 | Gnb1 |
| 1455878_at | 8.98 | 2700023E23Rik |
| 1450496_a_at | 8.74 | 2810433K01Rik |
| 1452912_at | 8.63 | 2600005O03Rik |
| 1434767_at | 8.59 | C79407 |
| 1448568_a_at | 8.56 | Slc20a1 |
| 1456653_a_at | 8.56 | Mthfd1l |
| 1429642_at | 8.37 | Anubl1 |
| 1419943_s_at | 8.30 | Ccnb1 |
| 1453589_a_at | 8.26 | 6820431F20Rik |
| 1439377_x_at | 8.26 | Cdc20 |
| 1419029_at | 8.19 | Ero1l |
| 1436723_at | 8.18 | Cenpi |
| 1429527_a_at | 8.14 | LOC433328 /// LOC677340 /// Plscr1 |
| 1424971_at | 8.09 | Ccdc99 |
| 1452115_a_at | 8.09 | Plk4 |
| 1439695_a_at | 8.04 | Mphosph1 |
| 1428104_at | 7.86 | Tpx2 |
| 1421546_a_at | 7.85 | Racgap1 |
| 1418334_at | 7.83 | Dbf4 |
| 1455488_at | 7.81 | 6230416J20Rik |
| 1448314_at | 7.80 | Cdc2a |
| 1451358_a_at | 7.78 | Racgap1 |
| 1416258_at | 7.77 | Tk1 |
| 1418281_at | 7.77 | Rad51 |
| 1422430_at | 7.72 | Fignl1 |
| 1460247_a_at | 7.69 | Skp2 |
| 1452314_at | 7.68 | Kif11 |
| 1429172_a_at | 7.55 | Ncapg |
| 1432538_a_at | 7.46 | Rfc3 |
| 1448205_at | 7.43 | Ccnb1 /// Ccnb1-rs1 |
| 1424629_at | 7.40 | Brca1 |
| 1455160_at | 7.39 | 2610203C20Rik |
| 1450920_at | 7.35 | Ccnb2 |
| 1451920_a_at | 7.22 | Rfc1 |
| 1424143_a_at | 7.15 | Cdt1 |
| 1453181_x_at | 7.14 | Plscr1 |
| 1452954_at | 7.07 | Ube2c |
| 1453107_s_at | 6.98 | 4933413G19Rik /// Foxm1 /// Pebp1 |
| 1417139_at | 6.97 | Dsn1 |
| 1437187_at | 6.96 | E2f7 /// LOC639365 |
| 1447363_s_at | 6.90 | Bub1b |
| 1429244_at | 6.85 | 2610524H06Rik |
| 1429156_at | 6.84 | 2610036L11Rik |
| 1424144_at | 6.75 | Cdt1 |
| 1417458_s_at | 6.73 | Cks2 /// LOC100039474 /// LOC100044750 /// LOC100044764 |
| 1422028_a_at | 6.64 | Ets1 |
| 1437033_a_at | 6.58 | Skp2 |
| 1437251_at | 6.51 | Cdca2 |
| 1436808_x_at | 6.47 | Mcm5 |
| 1415811_at | 6.47 | Uhrf1 |
| 1423524_at | 6.47 | Mastl |
| 1429660_s_at | 6.41 | Smc2 |
| 1445824_at | 6.40 | Zfp458 |
| 1416514_a_at | 6.36 | Fscn1 |
| 1456857_at | 6.36 | 1500011B03Rik |
| 1440882_at | 6.35 | Lrp8 |
| 1417911_at | 6.34 | Ccna2 |
| 1454694_a_at | 6.33 | Top2a |
| 1435005_at | 6.33 | Cenpe |
| 1426002_a_at | 6.28 | Cdc7 |
| 1416664_at | 6.27 | Cdc20 |
| 1430574_at | 6.26 | Cdkn3 |
| 1416309_at | 6.26 | Nusap1 |
| 1423520_at | 6.24 | Lmnb1 |
| 1417910_at | 6.21 | Ccna2 |
| 1450842_a_at | 6.20 | Cenpa |
| 1433893_s_at | 6.20 | Spag5 |
| 1416575_at | 6.17 | Cdc45l |
| 1437370_at | 6.15 | Sgol2 |
| 1435575_at | 6.14 | Kntc1 |
| 1418969_at | 6.12 | Skp2 |
| 1448627_s_at | 6.12 | Pbk |
| 1452040_a_at | 6.11 | Cdca3 |
| 1435135_at | 6.10 | Aadacl1 |
| 1422663_at | 6.08 | Orc1l |
| 1450157_a_at | 6.07 | Hmmr |
| 1422460_at | 6.03 | Mad2l1 |
| 1423809_at | 5.97 | Tcf19 |
| 1451928_a_at | 5.94 | Rad18 |
| 1460378_a_at | 5.92 | Tes |
| 1452305_s_at | 5.91 | Cenpn |
| 1439436_x_at | 5.89 | Incenp |
| 1428543_at | 5.87 | Ppat |
| 1417457_at | 5.84 | Cks2 /// LOC100039474 /// LOC100044764 |
| 1453775_at | 5.84 | 4921505C17Rik |
| 1437658_a_at | 5.81 | Snhg1 |
| 1435306_a_at | 5.80 | Kif11 |
| 1419838_s_at | 5.79 | Plk4 |
| 1426652_at | 5.78 | LOC100045677 /// Mcm3 |
| 1436707_x_at | 5.76 | Ncaph |
| 1458585_at | 5.74 | --- |
| 1451346_at | 5.72 | Mtap |
| 1456280_at | 5.71 | Clspn |
| 1448635_at | 5.68 | Smc2 |
| 1436708_x_at | 5.67 | Mcm4 |
| 1458586_at | 5.66 | --- |
| 1423774_a_at | 5.62 | Prc1 |
| 1448650_a_at | 5.62 | Pole |
| 1424991_s_at | 5.61 | Tyms /// Tyms-ps |
| 1433408_a_at | 5.59 | Mcm10 |
| 1417506_at | 5.53 | Gmnn |
| 1448953_at | 5.51 | Blm |
| 1439510_at | 5.50 | Sgol1 |
| 1445427_at | 5.49 | D11Ertd80e |
| 1424118_a_at | 5.47 | Spc25 |
| 1429294_at | 5.45 | Trip13 |
| 1428304_at | 5.45 | Esco2 |
| 1452226_at | 5.45 | LOC100047340 /// Rcc2 |
| 1427541_x_at | 5.44 | Hmmr |
| 1433892_at | 5.42 | Spag5 |
| 1422016_a_at | 5.41 | Cenph |
| 1416961_at | 5.40 | Bub1b |
| 1460403_at | 5.34 | Psip1 |
| 1423700_at | 5.32 | Rfc3 |
| 1436847_s_at | 5.31 | Cdca8 |
| 1449060_at | 5.29 | Kif2c /// LOC631653 |
| 1455355_at | 5.29 | 6030408C04Rik |
| 1425815_a_at | 5.29 | Hmmr |
| 1428481_s_at | 5.26 | Cdca8 |
| 1416698_a_at | 5.26 | Cks1b |
| 1417587_at | 5.22 | Timeless |
| 1421284_at | 5.21 | Pign |
| 1460353_at | 5.20 | Tmem48 |
| 1417445_at | 5.19 | Ndc80 |
| 1436174_at | 5.18 | Atad2 |
| 1424766_at | 5.16 | Ercc6l |
| 1430193_at | 5.16 | Casc5 |
| 1423318_at | 5.14 | Rad18 |
| 1427147_at | 5.14 | F730047E07Rik |
| 1423310_at | 5.09 | Tpbg |
| 1422768_at | 5.07 | Syncrip |
| 1426569_a_at | 4.99 | Frk |
| 1417705_at | 4.99 | LOC100046081 /// Otub1 |
| 1450862_at | 4.92 | Rad54l |
| 1423877_at | 4.88 | Chaf1b |
| 1453189_at | 4.87 | Ube2i |
| 1453745_at | 4.87 | 2700038G22Rik |
| 1422513_at | 4.85 | Ccnf |
| 1453683_a_at | 4.84 | Cep55 |
| 1422979_at | 4.82 | Suv39h2 |
| 1435597_at | 4.78 | Atad5 |
| 1451128_s_at | 4.75 | Kif22 |
| 1437716_x_at | 4.75 | Kif22 |
| 1429270_a_at | 4.75 | Syce2 |
| 1449140_at | 4.72 | Nudcd2 |
| 1438750_at | 4.70 | Atrx |
| 1422054_a_at | 4.63 | Skil |
| 1431235_at | 4.55 | 1110061A14Rik |
| 1415859_at | 4.52 | Eif3c |
| 1420707_a_at | 4.48 | Traip |
| 1454744_at | 4.46 | F630043A04Rik |
| 1422706_at | 4.42 | Tmepai |
| 1451602_at | 4.42 | Snx6 |
| 1451839_a_at | 4.39 | Pde7a |
| 1451246_s_at | 4.39 | Aurkb |
| 1419130_at | 4.39 | Adat2 |
| 1423417_at | 4.35 | Smarcc1 |
| 1450692_at | 4.33 | Kif4 |
| 1452983_at | 4.31 | Cep57 |
| 1428518_at | 4.30 | Mlf1ip |
| 1429121_at | 4.26 | Spopl |
| 1445689_at | 4.26 | --- |
| 1434079_s_at | 4.24 | Mcm2 |
| 1443962_at | 4.19 | 1110029I05Rik /// Tfdp2 |
| 1427141_at | 4.17 | 2700099C18Rik |
| 1452917_at | 4.16 | Rfc5 |
| 1449661_at | 4.12 | Suz12 |
| 1420909_at | 4.12 | Vegfa |
| 1424107_at | 4.10 | Kif18a |
| 1427379_at | 4.09 | --- |
| 1428480_at | 4.08 | Cdca8 |
| 1439095_at | 4.08 | Sfrs11 |
| 1418227_at | 4.06 | Orc2l |
| 1422462_at | 4.05 | Ube2t |
| 1436222_at | 4.05 | Gas5 |
| 1458007_at | 4.04 | Myo1b |
| 1434748_at | 4.01 | Ckap2 |
| 1426612_at | 3.94 | Tipin |
| 1433242_at | 3.94 | 5830415B17Rik |
| 1451417_at | 3.93 | Brca1 |
| 1438076_at | 3.91 | Rpl30 |
| 1415860_at | 3.89 | Kpna2 /// LOC100039592 /// LOC100043906 /// LOC100046251 |
| 1423620_at | 3.89 | Cenpq |
| 1448140_at | 3.88 | Ciapin1 |
| 1453226_at | 3.88 | 3000004C01Rik |
| 1459350_at | 3.85 | --- |
| 1440314_at | 3.84 | --- |
| 1432393_a_at | 3.84 | Thg1l |
| 1448191_at | 3.81 | Plk1 |
| 1433966_x_at | 3.80 | Asns |
| 1425166_at | 3.80 | Rbl1 |
| 1457744_at | 3.78 | Ddx46 |
| 1416031_s_at | 3.78 | Mcm7 |
| 1452241_at | 3.77 | Topbp1 |
| 1423525_at | 3.75 | Mastl |
| 1446838_at | 3.71 | Atad1 |
| 1460448_s_at | 3.71 | Ttc14 |
| 1456032_x_at | 3.69 | EG666634 /// H2afz |
| 1428968_at | 3.69 | Cep57 |
| 1460447_at | 3.67 | Pus7l |
| 1421731_a_at | 3.66 | Fen1 |
| 1455834_x_at | 3.66 | Tacc3 |
| 1423714_at | 3.65 | Asf1b |
| 1440498_at | 3.65 | --- |
| 1442109_at | 3.64 | --- |
| 1428593_at | 3.63 | 1700029F09Rik |
| 1428727_at | 3.63 | Cep192 |
| 1436728_s_at | 3.62 | Rtel1 |
| 1416563_at | 3.59 | Ctps |
| 1454703_x_at | 3.56 | Snhg1 |
| 1454952_s_at | 3.53 | Ncapd3 |
| 1438673_at | 3.50 | Slc4a7 |
| 1420081_s_at | 3.50 | D2Ertd750e |
| 1428483_a_at | 3.49 | 2610039C10Rik |
| 1422027_a_at | 3.47 | Ets1 |
| 1437549_at | 3.47 | 2810408I11Rik |
| 1429326_at | 3.46 | Cenpl |
| 1455905_at | 3.45 | 2610507B11Rik |
| 1427437_at | 3.43 | --- |
| 1439266_a_at | 3.43 | Polr3k |
| 1438051_at | 3.42 | Ttc14 |
| 1460677_at | 3.40 | Spats2 |
| 1419076_a_at | 3.36 | Brca2 |
| 1452258_at | 3.34 | Phf20 |
| 1434316_at | 3.33 | Chsy1 /// LOC100047167 |
| 1426554_a_at | 3.33 | Pgam1 |
| 1426739_at | 3.33 | Donson |
| 1430147_a_at | 3.32 | Josd3 |
| 1427404_x_at | 3.32 | EG433182 /// Eno1 /// LOC100044223 /// LOC100045967 /// LOC100047043 /// LOC100047882 |
| 1425197_at | 3.32 | Ptpn2 |
| 1460672_at | 3.31 | 2410002F23Rik |
| 1436349_at | 3.26 | 2700094K13Rik |
| 1416962_at | 3.26 | Rcc1 |
| 1457081_at | 3.24 | D630038D15Rik |
| 1457669_x_at | 3.23 | Rfc2 |
| 1438434_at | 3.21 | Arhgap11a |
| 1429895_at | 3.20 | 2310010G23Rik |
| 1431506_s_at | 3.19 | EG665989 /// LOC433064 /// LOC629952 /// LOC666411 /// Ppih |
| 1449877_s_at | 3.15 | Kifc1 /// LOC100042970 /// LOC100044006 /// LOC100044746 |
| 1429658_a_at | 3.15 | Smc2 |
| 1432013_a_at | 3.13 | 2610016C23Rik |
| 1430271_x_at | 3.13 | Josd3 /// LOC666781 |
| 1424136_a_at | 3.12 | EG665989 /// EG667598 /// LOC433064 /// LOC624822 /// Ppih |
| 1440391_at | 3.11 | LOC638038 |
| 1424300_at | 3.10 | Gemin6 |
| 1429810_at | 3.10 | 4921505C17Rik |
| 1459957_at | 3.10 | --- |
| 1415878_at | 3.10 | Rrm1 |
| 1419023_x_at | 3.09 | EG433182 /// Eno1 /// LOC100044223 /// LOC100045967 /// LOC100047043 /// LOC100047882 |
| 1435057_x_at | 3.09 | Polr1e |
| 1436247_at | 3.07 | Ints2 |
| 1441931_x_at | 3.07 | Gss |
| 1424142_at | 3.07 | Ikbkap |
| 1424156_at | 3.07 | Rbl1 |
| 1452454_at | 3.05 | Sdad1 |
| 1440875_a_at | 3.05 | Rsad1 |
| 1429395_at | 3.05 | Gstcd |
| 1418566_s_at | 3.04 | Nudcd2 |
| 1459307_at | 3.03 | --- |
| 1440553_at | 3.02 | Mecr |
| 1429048_at | 3.00 | Bloc1s2 |
| 1423919_at | 3.00 | BC023882 |
| 1439394_x_at | 3.00 | Cdc20 |
| 1440972_at | 2.96 | Nsd1 |
| 1418335_a_at | 2.95 | Dph1 /// RP23-143A14.5 |
| 1427105_at | 2.95 | Cenpn |
| 1450983_at | 2.91 | Akap8 |
| 1415913_at | 2.90 | EG625298 /// EG628061 /// LOC100039924 /// LOC100043695 /// LOC100044992 /// LOC100048340 /// LOC100048495 /// LOC637251 /// LOC668239 /// Rps13 |
| 1429665_at | 2.90 | 6230416J20Rik |
| 1419022_a_at | 2.89 | EG433182 /// Eno1 /// LOC100044223 /// LOC100045967 /// LOC545568 |
| 1426609_at | 2.88 | Dis3 |
| 1455726_at | 2.88 | Gm71 /// LOC100048447 |
| 1417351_a_at | 2.87 | LOC100047155 /// Snrpa1 |
| 1457509_at | 2.85 | Sbno1 |
| 1430987_s_at | 2.84 | Wbp11 |
| 1456541_x_at | 2.84 | Atad3a |
| 1426447_at | 2.83 | Nup35 |
| 1449200_at | 2.82 | Nup155 |
| 1455218_at | 2.82 | 6330503K22Rik |
| 1418906_at | 2.79 | Nubp1 |
| 1437497_a_at | 2.78 | Hsp90aa1 |
| 1459371_at | 2.78 | --- |
| 1452635_x_at | 2.77 | Josd3 |
| 1429242_at | 2.77 | 1110054O05Rik |
| 1416801_at | 2.75 | Trpm7 |
| 1435451_at | 2.74 | Hel308 |
| 1438053_at | 2.73 | Tfg |
| 1445169_at | 2.66 | --- |
| 1416448_at | 2.66 | Itpa |
| 1417125_at | 2.66 | Ahcy |
| 1426797_at | 2.66 | 2700094F01Rik |
| 1426676_s_at | 2.66 | Tomm70a |
| 1434692_at | 2.66 | 1110034B05Rik |
| 1452612_at | 2.63 | Zfp294 |
| 1439270_x_at | 2.62 | LOC100045999 /// LOC640204 /// Ran |
| 1452836_at | 2.59 | Lpin2 |
| 1436848_x_at | 2.58 | Impa1 |
| 1434317_s_at | 2.55 | Tex10 |
| 1453314_x_at | 2.54 | 2610039C10Rik |
| 1436930_x_at | 2.52 | EG623818 /// Hmbs |
| 1424589_s_at | 2.52 | Rnpc3 |
| 1426327_s_at | 2.51 | Zfp91 /// Zfp91-cntf |
| 1427269_at | 2.51 | Sfrs11 |
| 1426839_at | 2.49 | Pold3 |
| 1455818_at | 2.49 | 4930427A07Rik |
| 1417095_a_at | 2.45 | Hspa14 |
| 1436209_at | 2.45 | Dnajc16 |
| 1434660_at | 2.44 | Alkbh1 |
| 1455185_s_at | 2.44 | Phf16 |
| 1426469_a_at | 2.43 | Tbp |
| 1425460_at | 2.43 | Mtmr2 |
| 1451163_at | 2.41 | Tinf2 |
| 1437565_a_at | 2.39 | Gnl2 /// LOC633966 |
| 1448855_at | 2.37 | Rassf1 |
| 1443988_at | 2.34 | Rbm39 |
| 1417681_at | 2.33 | Nudt21 |
| 1437457_a_at | 2.32 | Mtpn |
| 1456730_x_at | 2.31 | Actl6a |
| 1436454_x_at | 2.27 | Fen1 |
| 1423303_at | 2.23 | Paxip1 |
| 1436022_at | 2.21 | Endogl1 |
| 1451641_at | 2.21 | Dbr1 |
| 1423826_at | 2.20 | Noc4l |
| 1424119_at | 2.18 | Prkab1 |
| 1454968_at | 2.17 | 1110034A24Rik |
| 1446007_at | 2.17 | --- |
| 1433960_at | 2.16 | Isg20l2 |
| 1426386_at | 2.16 | Rpl7l1 |
| 1416048_at | 2.15 | Phc2 |
| 1451408_at | 2.12 | Trub2 |
| 1421122_at | 2.12 | Cbll1 |
| 1439464_s_at | 2.12 | Tex10 |
| 1433567_at | 2.10 | Gmps |
| 1426548_a_at | 2.08 | Atpbd4 |
| 1441737_s_at | 2.06 | Rassf1 |
| 1421906_at | 1.94 | Med1 |
| 1442106_at | 1.87 | Fancm |
| 1440061_at | 1.86 | Rbx1 |
| 1428248_at | 1.81 | Nfx1 |
| 1426836_s_at | 1.78 | Metap1 |
| 1426854_a_at | 1.75 | BC085271 /// LOC100047898 /// Set |
| 1454818_at | 1.70 | Gmeb2 |
