# Supplementary material for: Gene expression profiling of mouse p53-deficient epidermal carcinoma defines molecular determinants of human cancer malignancy
Source: Mol Cancer. 2010 Jul 14;9:193. doi: 10.1186/1476-4598-9-193 (PMC2913987; doi:10.1186/1476-4598-9-193)
Supplement: Additional file 3 — 311 probesets underexpressed in mouse p53-tumors. The table includes the probesets IDs, fold change and gene symbol corresponding to the underexpressed Affymetrix probesets in the mouse tumors of the training dataset. [file 1476-4598-9-193-S3.DOC]

**Additional file 3.** 311 probesets underexpressed in mouse p53-tumors.

| **ProbesetID** | **Fold Change** | **Gene Symbol** |
| --- | --- | --- |
| 1427026_at | -375.82 | Myh4 |
| 1427868_x_at | -324.67 | Myh1 |
| 1417464_at | -300.83 | Tnnc2 |
| 1450118_a_at | -261.96 | Tnnt3 |
| 1433923_at | -230.95 | Krt77 |
| 1417653_at | -229.18 | Pvalb |
| 1422651_at | -227.02 | Adipoq |
| 1427520_a_at | -223.00 | Myh1 |
| 1419312_at | -218.04 | Atp2a1 |
| 1427445_a_at | -217.89 | Ttn |
| 1452651_a_at | -216.69 | Myl1 |
| 1427446_s_at | -215.16 | Ttn |
| 1455736_at | -200.78 | Mybpc2 |
| 1417867_at | -197.77 | Cfd |
| 1449434_at | -196.01 | Car3 |
| 1417614_at | -191.48 | Ckm |
| 1418155_at | -178.78 | Myot |
| 1448602_at | -166.36 | Pygm |
| 1418095_at | -160.98 | Smpx |
| 1428722_at | -160.83 | Ckmt2 |
| 1435355_at | -151.06 | Neb |
| 1460256_at | -146.79 | Car3 |
| 1457435_x_at | -144.78 | Myom2 |
| 1429565_s_at | -133.24 | Lce1m |
| 1417889_at | -129.59 | Apobec2 |
| 1448371_at | -129.23 | Mylpf |
| 1416889_at | -117.31 | Tnni2 |
| 1426144_x_at | -113.16 | Trdn |
| 1455645_at | -101.46 | 8030451F13Rik |
| 1427735_a_at | -100.34 | Acta1 |
| 1427306_at | -95.82 | Ryr1 |
| 1416752_at | -95.36 | Ldb3 |
| 1415994_at | -95.07 | Cyp2e1 |
| 1420693_at | -91.31 | Myom1 |
| 1424737_at | -90.93 | Thrsp |
| 1448327_at | -86.91 | Actn2 |
| 1418677_at | -83.31 | Actn3 |
| 1428899_at | -76.55 | Tmem182 |
| 1438936_s_at | -75.96 | Ang |
| 1449500_at | -75.11 | Serpinb7 |
| 1427312_at | -74.54 | Cmya5 |
| 1448792_a_at | -74.50 | Cyp2f2 |
| 1426143_at | -72.26 | Trdn |
| 1417951_at | -70.41 | Eno3 |
| 1451203_at | -69.86 | Mb |
| 1426142_a_at | -62.88 | Trdn |
| 1422644_at | -61.60 | LOC100045042 /// Sh3bgr |
| 1449356_at | -61.10 | Asb5 |
| 1449015_at | -59.22 | Retnla |
| 1436453_at | -57.57 | BB144871 |
| 1418373_at | -56.62 | Pgam2 |
| 1435813_at | -55.63 | Mypn |
| 1449218_at | -54.63 | Cox8b |
| 1460202_at | -53.22 | Myoz1 |
| 1450917_at | -53.14 | Myom2 |
| 1433783_at | -52.09 | Ldb3 |
| 1444494_at | -49.97 | Kbtbd10 |
| 1452345_at | -49.80 | Lmod2 |
| 1433532_a_at | -49.18 | Mbp |
| 1435370_a_at | -48.47 | Ces3 |
| 1452879_at | -47.86 | Synpo2 |
| 1449398_at | -46.63 | Rpl3l |
| 1417273_at | -46.56 | Pdk4 |
| 1456508_at | -46.50 | A030001H23Rik |
| 1447870_x_at | -46.34 | 1110002E22Rik |
| 1449969_at | -46.09 | Tmod4 |
| 1423477_at | -44.86 | Zic1 |
| 1419056_at | -44.74 | Rtn2 |
| 1460318_at | -44.08 | Csrp3 |
| 1452260_at | -43.88 | Cidec |
| 1416468_at | -41.04 | Aldh1a1 |
| 1417626_at | -40.91 | Pde4dip |
| 1417607_at | -40.34 | Cox6a2 |
| 1418709_at | -39.46 | Cox7a1 |
| 1439658_at | -38.02 | Lmod3 |
| 1421253_at | -37.61 | Nrap |
| 1438937_x_at | -36.14 | Ang |
| 1441667_s_at | -34.87 | Smyd1 |
| 1423145_a_at | -34.75 | Tcap |
| 1449178_at | -34.15 | Pdlim3 |
| 1438609_x_at | -34.11 | Tnni2 |
| 1436332_at | -33.69 | Hspb6 |
| 1436867_at | -33.07 | Srl |
| 1420442_at | -32.14 | Cacna1s |
| 1434893_at | -31.82 | Atp1a2 |
| 1426731_at | -31.52 | Des |
| 1447657_s_at | -30.98 | Synpo2l |
| 1450798_at | -30.76 | Tnxb |
| 1416023_at | -29.76 | Fabp3 |
| 1438467_at | -28.75 | Mgl2 |
| 1438175_x_at | -28.30 | Myom2 |
| 1448842_at | -27.00 | Cdo1 |
| 1429223_a_at | -26.95 | Hfe2 |
| 1420582_at | -26.86 | Cd209e |
| 1416455_a_at | -26.78 | Cryab |
| 1418769_at | -26.08 | Myoz2 |
| 1418595_at | -25.89 | S3-12 |
| 1429463_at | -25.59 | Prkaa2 |
| 1451372_a_at | -25.58 | Art1 |
| 1459030_at | -25.22 | Bbox1 |
| 1421092_at | -25.01 | Serpina12 |
| 1456228_x_at | -24.46 | Mbp |
| 1419618_at | -23.88 | Bbox1 |
| 1417765_a_at | -23.64 | Amy1 |
| 1419739_at | -23.18 | Tpm2 |
| 1434449_at | -23.09 | Aqp4 |
| 1421374_a_at | -22.90 | Fxyd1 |
| 1418601_at | -22.63 | Aldh1a7 |
| 1427867_at | -22.58 | --- |
| 1443997_at | -22.06 | Gprin2 |
| 1443906_at | -21.84 | Cd55 |
| 1418314_a_at | -21.79 | A2bp1 |
| 1422153_a_at | -21.74 | Asb11 |
| 1426172_a_at | -21.73 | Cd209a |
| 1425275_at | -21.37 | Asph |
| 1416225_at | -21.31 | Adh1 |
| 1423238_at | -21.16 | Itgb1bp2 |
| 1434008_at | -21.16 | Scn4b |
| 1449383_at | -20.93 | Adssl1 |
| 1427371_at | -20.85 | Abca8a |
| 1434766_at | -19.99 | --- |
| 1427465_at | -19.64 | Atp1a2 |
| 1419646_a_at | -19.50 | Mbp |
| 1449081_at | -19.50 | Ces3 |
| 1419145_at | -19.04 | Smtnl1 |
| 1419440_at | -18.81 | Trim54 |
| 1443783_x_at | -18.75 | H2-Aa |
| 1453059_at | -17.76 | 2310046A06Rik |
| 1417025_at | -17.57 | H2-Eb1 |
| 1424177_at | -17.48 | Tmem38a |
| 1426183_a_at | -17.18 | Cd209d |
| 1418062_at | -17.02 | Eef1a2 |
| 1436201_x_at | -16.87 | Mbp |
| 1419150_at | -16.19 | Myf6 |
| 1434927_at | -15.86 | Hspb7 |
| 1417979_at | -15.56 | Tnmd |
| 1418697_at | -15.28 | Inmt |
| 1424531_a_at | -15.21 | Tcea3 |
| 1455466_at | -14.92 | Gpr133 |
| 1449918_at | -14.84 | Cd209g |
| 1423405_at | -14.77 | Timp4 |
| 1435399_at | -14.53 | 2310068J10Rik |
| 1416780_at | -14.41 | Pfkm |
| 1452590_a_at | -14.30 | LOC100039175 /// LOC100039246 /// Plac9 |
| 1449466_at | -13.75 | Clec3b |
| 1460591_at | -13.68 | Esr1 |
| 1455610_at | -13.56 | Dmn |
| 1450884_at | -13.52 | Cd36 |
| 1424393_s_at | -13.48 | Adhfe1 |
| 1448507_at | -13.23 | Efhd1 |
| 1436939_at | -13.23 | Unc45b |
| 1454651_x_at | -13.22 | Mbp |
| 1448926_at | -12.94 | Hoxa5 |
| 1435371_x_at | -12.89 | Ces3 |
| 1439505_at | -12.81 | Clic5 |
| 1449577_x_at | -12.77 | Tpm2 |
| 1448154_at | -12.18 | Ndrg2 |
| 1420551_at | -11.80 | 2310039E09Rik |
| 1448249_at | -11.73 | Gpd1 |
| 1425505_at | -11.27 | Mylk |
| 1423253_at | -11.00 | Mpz |
| 1435312_at | -10.99 | Paqr7 |
| 1418028_at | -10.96 | Dct |
| 1449396_at | -10.86 | Aoc3 |
| 1456895_at | -10.79 | Cd209b |
| 1460674_at | -10.76 | Paqr7 |
| 1448962_at | -10.73 | Myh11 |
| 1460242_at | -10.68 | Cd55 |
| 1436098_at | -10.50 | Bche |
| 1416697_at | -10.37 | Dpp4 |
| 1451322_at | -10.37 | Cmbl |
| 1456741_s_at | -10.22 | Gpm6a |
| 1417168_a_at | -10.12 | Usp2 |
| 1460012_at | -10.01 | Wfdc3 |
| 1429236_at | -9.99 | Galntl2 |
| 1436044_at | -9.83 | Scn7a |
| 1449971_a_at | -9.80 | Cd209f |
| 1438431_at | -9.49 | Abcd2 |
| 1429783_at | -9.36 | Pdlim5 |
| 1451969_s_at | -9.24 | Parp3 |
| 1421254_a_at | -9.22 | Sgcg |
| 1425677_a_at | -9.10 | Ank1 |
| 1448825_at | -8.88 | Pdk2 |
| 1417066_at | -8.87 | Cabc1 |
| 1449088_at | -8.86 | Fbp2 |
| 1433720_s_at | -8.71 | Ndg2 |
| 1427201_at | -8.58 | Mustn1 |
| 1442226_at | -8.43 | Sema3e |
| 1419687_at | -8.36 | Macrod1 |
| 1426157_a_at | -8.35 | Cd209b |
| 1429598_at | -8.26 | 2310042D19Rik |
| 1433727_at | -8.00 | BC038479 |
| 1418762_at | -7.85 | Cd55 |
| 1441551_at | -7.72 | Mypn |
| 1418952_at | -7.71 | Txlnb |
| 1425243_at | -7.69 | Cd207 |
| 1436737_a_at | -7.66 | Sorbs1 |
| 1421144_at | -7.48 | Rpgrip1 |
| 1442769_at | -7.41 | 8030451F13Rik |
| 1416007_at | -7.34 | Satb1 |
| 1425826_a_at | -7.09 | Sorbs1 |
| 1450723_at | -7.08 | Isl1 |
| 1422654_at | -6.89 | Sgca |
| 1432517_a_at | -6.85 | Nnmt |
| 1428471_at | -6.77 | Sorbs1 |
| 1447946_at | -6.60 | Adam23 |
| 1418395_at | -6.56 | Slc47a1 |
| 1417027_at | -6.42 | Trim2 |
| 1435446_a_at | -6.36 | Chpt1 |
| 1418086_at | -6.27 | Ppp1r14a |
| 1429808_at | -6.23 | 1110020C03Rik |
| 1418100_at | -6.16 | A030009H04Rik |
| 1417787_at | -6.11 | Dkkl1 |
| 1434786_at | -6.08 | Ppp1r12b |
| 1457031_at | -6.05 | Fsd2 |
| 1453289_at | -6.04 | Eif2c4 |
| 1459646_at | -6.01 | Hs3st6 |
| 1435272_at | -5.98 | Itpkb |
| 1439708_at | -5.91 | Myom3 |
| 1435663_at | -5.68 | Esr1 |
| 1425274_at | -5.65 | Asph |
| 1438698_at | -5.47 | Tmem132c |
| 1418743_a_at | -5.47 | LOC100047138 /// Tesc |
| 1418804_at | -5.44 | Sucnr1 |
| 1415958_at | -5.36 | Slc2a4 |
| 1417169_at | -5.24 | Usp2 |
| 1440859_at | -5.10 | Akap6 |
| 1436987_at | -5.10 | 5430433G21Rik |
| 1435870_at | -5.07 | --- |
| 1416008_at | -5.03 | Satb1 |
| 1424408_at | -5.03 | Lims2 |
| 1450717_at | -4.98 | Ang |
| 1417680_at | -4.91 | Kcna5 |
| 1456087_at | -4.80 | Nfia |
| 1453355_at | -4.78 | Wnk2 |
| 1427177_at | -4.69 | Fyco1 |
| 1430368_s_at | -4.61 | 1700019D03Rik |
| 1419301_at | -4.61 | Fzd4 |
| 1433628_at | -4.51 | Coq10a |
| 1421091_at | -4.49 | Serpina12 |
| 1436224_at | -4.44 | Kif1c |
| 1427943_at | -4.44 | Acyp2 |
| 1434756_at | -4.41 | 5430421B17 |
| 1455091_at | -4.40 | 3222402P14Rik |
| 1454078_a_at | -4.34 | Gal3st1 |
| 1426460_a_at | -4.21 | Ugp2 |
| 1434511_at | -4.20 | Phkb |
| 1422245_a_at | -4.20 | Mrvi1 |
| 1460244_at | -4.19 | Upb1 |
| 1460123_at | -4.18 | Gpr1 |
| 1419554_at | -4.17 | Cd47 |
| 1424308_at | -4.16 | Slc24a3 |
| 1450725_s_at | -4.15 | Car14 |
| 1430979_a_at | -4.11 | Prdx2 |
| 1451326_at | -4.11 | Abhd14b |
| 1437211_x_at | -4.04 | Elovl5 |
| 1453486_a_at | -3.86 | Scube2 |
| 1423860_at | -3.85 | Ptgds |
| 1455739_at | -3.81 | EG245190 |
| 1436841_at | -3.77 | B230380D07Rik |
| 1453749_at | -3.74 | 2610507I01Rik |
| 1420827_a_at | -3.74 | Ccng1 |
| 1419054_a_at | -3.70 | Ptpn21 |
| 1449620_s_at | -3.62 | D16Wsu65e |
| 1420444_at | -3.56 | Slc22a3 |
| 1433557_at | -3.48 | Cbx7 |
| 1452714_at | -3.46 | Tanc1 |
| 1454171_x_at | -3.32 | 9530053H05Rik |
| 1450017_at | -3.29 | Ccng1 |
| 1444998_at | -3.27 | --- |
| 1452929_at | -3.27 | Clip1 |
| 1460213_at | -3.25 | Golga4 |
| 1456569_x_at | -3.24 | Gsn |
| 1434454_at | -3.20 | D16Wsu65e |
| 1416838_at | -3.19 | Mut |
| 1453752_at | -3.04 | Rpl17 |
| 1448491_at | -3.00 | Ech1 |
| 1416816_at | -2.98 | Nek7 |
| 1457724_at | -2.92 | Ctsl |
| 1421867_at | -2.91 | Nr3c1 |
| 1428464_at | -2.89 | Ndufa3 |
| 1442508_at | -2.89 | 4933404M19Rik |
| 1419179_at | -2.89 | Txnl4a |
| 1447882_x_at | -2.88 | Ddx54 |
| 1423859_a_at | -2.86 | Ptgds |
| 1452790_x_at | -2.82 | Ndufa3 |
| 1436621_at | -2.73 | LOC100047169 /// RP23-157O10.7 |
| 1415966_a_at | -2.57 | Ndufv1 |
| 1436338_at | -2.53 | --- |
| 1434485_a_at | -2.49 | Ugp2 |
| 1422731_at | -2.45 | Limd1 |
| 1451312_at | -2.44 | Ndufs7 |
| 1416425_at | -2.41 | Pex19 |
| 1435113_x_at | -2.40 | Stmn3 |
| 1423498_at | -2.38 | Aldoart2 |
| 1451999_at | -2.32 | Ldb3 |
| 1439688_at | -2.31 | Fbln1 |
| 1452055_at | -2.25 | Ctdsp1 /// LOC100047249 |
| 1415984_at | -2.23 | Acadm |
| 1435008_at | -2.23 | Slc9a6 |
| 1456385_x_at | -2.19 | Ubxd3 |
| 1418954_at | -2.09 | Camkk1 |
| 1424280_at | -1.90 | Mospd1 |
| 1441923_s_at | -1.90 | Edn3 |
| 1443503_at | -1.88 | Ihpk3 |
| 1445558_at | -1.85 | 5930430L01Rik |
| 1436102_at | -1.80 | Sec22c |
| 1422908_at | -1.79 | Atp1b4 |
| 1432635_a_at | -1.73 | Tloc1 |
| 1459260_at | -1.69 | --- |
| 1419510_at | -1.60 | Es22 |
