# Supplementary material for: Gene expression profiling of mouse p53-deficient epidermal carcinoma defines molecular determinants of human cancer malignancy
Source: Mol Cancer. 2010 Jul 14;9:193. doi: 10.1186/1476-4598-9-193 (PMC2913987; doi:10.1186/1476-4598-9-193)
Supplement: Additional file 4 — Significant overlaps of human-mapped overexpressed or underexpressed genes from tumors of p53-deficient mouse with human tumors using "Molecular Subtype Mutations" and "Clinical Outcome" analyses. The file includes a table with the Concept Name, gene number overlap and overlapping significance of the signature of p53-deficient mouse compared to human signatures with p53-mutations or with poor outcome, and also includes the bibliographic references of the human signatures. [file 1476-4598-9-193-S4.DOC]

**Additional file 4.** Significant overlaps of human-mapped overexpressed or underexpressed mouse p53-genes with human tumors with “Molecular Subtype Mutations” and “Clinical Outcome”.

|  | **Concept Name** | **Overlap** | **p-val** | **Odds Ratio** | **Expression** | **Reference** |
| --- | --- | --- | --- | --- | --- | --- |
| **Molecular Subtype Mutation** | Breast Carcinoma - TP53 Mutation - Top 5% Over-expressed (Ivshina Breast) | 98 | 1.52E-66 | 16.2 | Over-expressed | [1] |
|  | Ductal Breast Carcinoma - TP53 Mutation - Top 10% Over-expressed (Sorlie Breast) | 42 | 1.09E-12 | 4.6 | Over-expressed | [2] |
|  | Breast Cancer Cell Line - TP53 Mutation - Top 10% Over-expressed (Neve CellLine) | 58 | 3.03E-11 | 3.1 | Over-expressed | [3] |
|  | Ductal Breast Carcinoma - TP53 Mutation - Top 10% Over-expressed (Pollack Breast 2) | 35 | 2.81E-09 | 3.8 | Over-expressed | [4] |
|  | Lung Adenocarcinoma - TP53 Mutation - Top 10% Over-expressed (Ding Lung) | 129 | 3.20E-54 | 7.9 | Over-expressed | [5] |
|  | Bladder Urothelial Carcinoma - TP53 Mutation - Top 5% Over-expressed (Lindgren Bladder) | 45 | 3.05E-25 | 10.3 | Over-expressed | [6] |
|  | Hepatocellular Carcinoma - TP53 Mutation - Top 10% Over-expressed (Chiang Liver) | 87 | 2.98E-22 | 4.1 | Over-expressed | [7] |
|  | Ovarian Endometrioid Adenocarcinoma - TP53 Mutation - Top 10% Over-expressed (Hendrix Ovarian) | 61 | 8.88E-13 | 3.3 | Over-expressed | [8] |
|  | Melanoma Cell Line - TP53 Mutation - Top 10% Over-expressed (Johansson CellLine) | 66 | 5.19E-11 | 2.8 | Over-expressed | [9] |
|  | Mantle Cell Lymphoma - TP53 Deletion - Top 10% Over-expressed (Rosenwald Lymphoma 3) | 31 | 1.54E-07 | 3.5 | Over-expressed | [10] |
|  |  |  |  |  |  |  |
| **Clinical Outcome** | Ductal Breast Carcinoma Epithelia - Dead at 1 Year - Top 10% Over-expressed (Boersma Breast) | 110 | 3.97E-49 | 8.7 | Over-expressed | [11] |
|  | Breast Carcinoma - Dead at 5 Years - Top 10% Over-expressed (Pawitan Breast) | 121 | 1.54E-47 | 7.1 | Over-expressed | [12] |
|  | Breast Carcinoma - Dead at 5 Years - Top 5% Over-expressed (vandeVijver Breast) | 84 | 1.19E-45 | 10.1 | Over-expressed | [13] |
|  | Breast Carcinoma - Dead at 3 Years - Top 10% Over-expressed (Bild Breast) | 51 | 5.30E-15 | 4.5 | Over-expressed | [14] |
|  | Ductal Breast Carcinoma Stroma - Dead at 1 Year - Top 10% Over-expressed (Boersma Breast) | 57 | 9.37E-11 | 3 | Over-expressed | [11] |
|  | Invasive Ductal Breast Carcinoma - Dead at 5 Years - Top 5% Over-expressed (Sotiriou Breast 2) | 20 | 6.84E-07 | 4.3 | Over-expressed | [15] |
|  | Mantle Cell Lymphoma - Dead at 1 Year - Top 5% Over-expressed (Rosenwald Lymphoma 3) | 41 | 2.69E-26 | 14.1 | Over-expressed | [10] |
|  | Diffuse Large B-Cell Lymphoma - Dead at 1 Year - Top 10% Over-expressed (Dave Lymphoma 2) | 19 | 1.77E-06 | 4.6 | Over-expressed | [16] |
|  | Astrocytoma - Dead at 3 Years - Top 10% Over-expressed (Phillips Brain) | 139 | 6.05E-64 | 9.3 | Over-expressed | [17] |
|  | Glioblastoma - Dead at 1 Year - Top 1% Over-expressed (Liang Brain) | 21 | 3.44E-17 | 16.6 | Over-expressed | [18] |
|  | Anaplastic Oligodendroglioma - Dead at 1 Year - Top 10% Over-expressed (Freije Brain) | 73 | 1.77E-14 | 3.2 | Over-expressed | [19] |
|  | Glioblastoma - Dead at 1 Year - Top 10% Over-expressed (Nutt Brain) | 46 | 1.14E-11 | 3.8 | Over-expressed | [20] |
|  | Classic Medulloblastoma - Dead at 3 Years - Top 10% Over-expressed (Pomeroy Brain) | 28 | 2.05E-07 | 3.7 | Over-expressed | [21] |
|  | Infiltrating Bladder Urothelial Carcinoma - Dead at 1 Year - Top 10% Over-expressed (Blaveri Bladder 2) | 49 | 3.50E-16 | 5.2 | Over-expressed | [22] |
|  | Superficial Bladder Cancer - Dead at 1 Year - Top 10% Over-expressed (Blaveri Bladder 2) | 32 | 7.70E-07 | 3 | Over-expressed | [22] |
|  | Superficial Bladder Cancer - Dead at 1 Year - Top 1% Over-expressed (Dyrskjot Bladder 5) | 4 | 7.64E-06 | 123.8 | Over-expressed | [23] |
|  | Lung Adenocarcinoma - Dead at 5 Years - Top 5% Over-expressed (DirectorsChallenge Lung) | 94 | 9.02E-62 | 15 | Over-expressed | [24] |
|  | Lung Adenocarcinoma - Dead at 3 Years - Top 10% Over-expressed (Bild Lung) | 69 | 2.12E-12 | 2.9 | Over-expressed | [14] |
|  | Lung Adenocarcinoma - Dead at 1 Year - Top 10% Over-expressed (Beer Lung) | 25 | 2.05E-05 | 3 | Over-expressed | [25] |
|  | Papillary Renal Cell Carcinoma - Dead at 5 Years - Top 5% Over-expressed (Yang Renal) | 70 | 1.46E-29 | 6.6 | Over-expressed | [26] |
|  | Clear Cell Renal Cell Carcinoma - Dead at 5 Years - Top 10% Over-expressed (Zhao Renal) | 75 | 6.78E-20 | 4.2 | Over-expressed | [27] |
|  | Multiple Myeloma - Dead at 3 Years - Top 10% Over-expressed (Zhan Myeloma 2) | 149 | 1.76E-73 | 10.6 | Over-expressed | [28] |
|  | Multiple Myeloma - Dead at 1 Year - Top 10% Over-expressed (Carrasco Myeloma) | 72 | 7.29E-14 | 3.1 | Over-expressed | [29] |
|  | Peritoneal Serous Adenocarcinoma - Dead at 3 Years - Top 10% Over-expressed (Tothill Ovarian) | 67 | 1.82E-11 | 2.8 | Over-expressed | [30] |
|  | Ovarian Endometrioid Adenocarcinoma - Dead at 5 Years - Top 10% Over-expressed (Lu Ovarian) | 55 | 4.11E-07 | 2.3 | Over-expressed | [31] |
|  | Prostate Carcinoma - Dead at 5 Years - Top 5% Over-expressed (Nakagawa Prostate) | 9 | 4.13E-08 | 24.6 | Over-expressed | [32] |
|  | Diffuse Gastric Adenocarcinoma - Dead at 1 Year - Top 10% Over-expressed (Chen Gastric) | 48 | 1.61E-07 | 2.6 | Over-expressed | [33] |
|  | Head and Neck Squamous Cell Carcinoma - Dead at 1 Year - Top 10% Over-expressed (Cromer Head-Neck) | 47 | 2.87E-12 | 4 | Over-expressed | [34] |
|  |  |  |  |  |  |  |
| **Clinical Outcome** | Head and Neck Squamous Cell Carcinoma - Dead at 5 Years - Top 10% Under-expressed (Chung Head-Neck) | 43 | 2.28E-12 | 4.3 | Under-expressed | [35] |
|  | Breast Carcinoma - Dead at 5 Years - Top 10% Under-expressed (Pawitan Breast) | 54 | 1.67E-09 | 2.8 | Under-expressed | [12] |
|  | Astrocytoma - Dead at 1 Year - Top 10% Under-expressed (Phillips Brain) | 48 | 6.03E-07 | 2.4 | Under-expressed | [17] |
|  | Lung Adenocarcinoma - Dead at 1 Year - Top 10% Under-expressed (DirectorsChallenge Lung) | 40 | 7.80E-07 | 2.6 | Under-expressed | [24] |
|  | Germinal Center B-Cell-Like Diffuse Large B-Cell Lymphoma - Dead at 5 Years - Top 5% Under-expressed (Lenz Lymphoma) | 29 | 5.18E-06 | 2.8 | Under-expressed | [36] |
|  | Lung Adenocarcinoma - Dead at 3 Years - Top 10% Under-expressed (Garber Lung) | 30 | 2.37E-05 | 2.6 | Under-expressed | [37] |

**References**

1. Ivshina AV, George J, Senko O, Mow B, Putti TC, Smeds J, Lindahl T, Pawitan Y, Hall P, Nordgren H, et al: **Genetic reclassification of histologic grade delineates new clinical subtypes of breast cancer.** *Cancer Res* 2006, **66:**10292-10301.

2. Sorlie T, Perou CM, Tibshirani R, Aas T, Geisler S, Johnsen H, Hastie T, Eisen MB, van de Rijn M, Jeffrey SS, et al: **Gene expression patterns of breast carcinomas distinguish tumor subclasses with clinical implications.** *Proc Natl Acad Sci U S A* 2001, **98:**10869-10874.

3. Neve RM, Chin K, Fridlyand J, Yeh J, Baehner FL, Fevr T, Clark L, Bayani N, Coppe JP, Tong F, et al: **A collection of breast cancer cell lines for the study of functionally distinct cancer subtypes.** *Cancer Cell* 2006, **10:**515-527.

4. Pollack JR, Sorlie T, Perou CM, Rees CA, Jeffrey SS, Lonning PE, Tibshirani R, Botstein D, Borresen-Dale AL, Brown PO: **Microarray analysis reveals a major direct role of DNA copy number alteration in the transcriptional program of human breast tumors.** *Proc Natl Acad Sci U S A* 2002, **99:**12963-12968.

5. Ding L, Getz G, Wheeler DA, Mardis ER, McLellan MD, Cibulskis K, Sougnez C, Greulich H, Muzny DM, Morgan MB, et al: **Somatic mutations affect key pathways in lung adenocarcinoma.** *Nature* 2008, **455:**1069-1075.

6. Lindgren D, Liedberg F, Andersson A, Chebil G, Gudjonsson S, Borg A, Mansson W, Fioretos T, Hoglund M: **Molecular characterization of early-stage bladder carcinomas by expression profiles, FGFR3 mutation status, and loss of 9q.** *Oncogene* 2006, **25:**2685-2696.

7. Chiang DY, Villanueva A, Hoshida Y, Peix J, Newell P, Minguez B, LeBlanc AC, Donovan DJ, Thung SN, Sole M, et al: **Focal gains of VEGFA and molecular classification of hepatocellular carcinoma.** *Cancer Res* 2008, **68:**6779-6788.

8. Hendrix ND, Wu R, Kuick R, Schwartz DR, Fearon ER, Cho KR: **Fibroblast growth factor 9 has oncogenic activity and is a downstream target of Wnt signaling in ovarian endometrioid adenocarcinomas.** *Cancer Res* 2006, **66:**1354-1362.

9. Johansson P, Pavey S, Hayward N: **Confirmation of a BRAF mutation-associated gene expression signature in melanoma.** *Pigment Cell Res* 2007, **20:**216-221.

10. Rosenwald A, Wright G, Wiestner A, Chan WC, Connors JM, Campo E, Gascoyne RD, Grogan TM, Muller-Hermelink HK, Smeland EB, et al: **The proliferation gene expression signature is a quantitative integrator of oncogenic events that predicts survival in mantle cell lymphoma.** *Cancer Cell* 2003, **3:**185-197.

11. Boersma BJ, Reimers M, Yi M, Ludwig JA, Luke BT, Stephens RM, Yfantis HG, Lee DH, Weinstein JN, Ambs S: **A stromal gene signature associated with inflammatory breast cancer.** *Int J Cancer* 2008, **122:**1324-1332.

12. Pawitan Y, Bjohle J, Amler L, Borg AL, Egyhazi S, Hall P, Han X, Holmberg L, Huang F, Klaar S, et al: **Gene expression profiling spares early breast cancer patients from adjuvant therapy: derived and validated in two population-based cohorts.** *Breast Cancer Res* 2005, **7:**R953-964.

13. van de Vijver MJ, He YD, van't Veer LJ, Dai H, Hart AA, Voskuil DW, Schreiber GJ, Peterse JL, Roberts C, Marton MJ, et al: **A gene-expression signature as a predictor of survival in breast cancer.** *N Engl J Med* 2002, **347:**1999-2009.

14. Bild AH, Yao G, Chang JT, Wang Q, Potti A, Chasse D, Joshi MB, Harpole D, Lancaster JM, Berchuck A, et al: **Oncogenic pathway signatures in human cancers as a guide to targeted therapies.** *Nature* 2006, **439:**353-357.

15. Sotiriou C, Neo SY, McShane LM, Korn EL, Long PM, Jazaeri A, Martiat P, Fox SB, Harris AL, Liu ET: **Breast cancer classification and prognosis based on gene expression profiles from a population-based study.** *Proc Natl Acad Sci U S A* 2003, **100:**10393-10398.

16. Dave SS, Fu K, Wright GW, Lam LT, Kluin P, Boerma EJ, Greiner TC, Weisenburger DD, Rosenwald A, Ott G, et al: **Molecular diagnosis of Burkitt's lymphoma.** *N Engl J Med* 2006, **354:**2431-2442.

17. Phillips HS, Kharbanda S, Chen R, Forrest WF, Soriano RH, Wu TD, Misra A, Nigro JM, Colman H, Soroceanu L, et al: **Molecular subclasses of high-grade glioma predict prognosis, delineate a pattern of disease progression, and resemble stages in neurogenesis.** *Cancer Cell* 2006, **9:**157-173.

18. Liang Y, Diehn M, Watson N, Bollen AW, Aldape KD, Nicholas MK, Lamborn KR, Berger MS, Botstein D, Brown PO, Israel MA: **Gene expression profiling reveals molecularly and clinically distinct subtypes of glioblastoma multiforme.** *Proc Natl Acad Sci U S A* 2005, **102:**5814-5819.

19. Freije WA, Castro-Vargas FE, Fang Z, Horvath S, Cloughesy T, Liau LM, Mischel PS, Nelson SF: **Gene expression profiling of gliomas strongly predicts survival.** *Cancer Res* 2004, **64:**6503-6510.

20. Nutt CL, Mani DR, Betensky RA, Tamayo P, Cairncross JG, Ladd C, Pohl U, Hartmann C, McLaughlin ME, Batchelor TT, et al: **Gene expression-based classification of malignant gliomas correlates better with survival than histological classification.** *Cancer Res* 2003, **63:**1602-1607.

21. Pomeroy SL, Tamayo P, Gaasenbeek M, Sturla LM, Angelo M, McLaughlin ME, Kim JY, Goumnerova LC, Black PM, Lau C, et al: **Prediction of central nervous system embryonal tumour outcome based on gene expression.** *Nature* 2002, **415:**436-442.

22. Blaveri E, Simko JP, Korkola JE, Brewer JL, Baehner F, Mehta K, Devries S, Koppie T, Pejavar S, Carroll P, Waldman FM: **Bladder cancer outcome and subtype classification by gene expression.** *Clin Cancer Res* 2005, **11:**4044-4055.

23. Dyrskjot L, Zieger K, Real FX, Malats N, Carrato A, Hurst C, Kotwal S, Knowles M, Malmstrom PU, de la Torre M, et al: **Gene expression signatures predict outcome in non-muscle-invasive bladder carcinoma: a multicenter validation study.** *Clin Cancer Res* 2007, **13:**3545-3551.

24. Shedden K, Taylor JM, Enkemann SA, Tsao MS, Yeatman TJ, Gerald WL, Eschrich S, Jurisica I, Giordano TJ, Misek DE, et al: **Gene expression-based survival prediction in lung adenocarcinoma: a multi-site, blinded validation study.** *Nat Med* 2008, **14:**822-827.

25. Beer DG, Kardia SL, Huang CC, Giordano TJ, Levin AM, Misek DE, Lin L, Chen G, Gharib TG, Thomas DG, et al: **Gene-expression profiles predict survival of patients with lung adenocarcinoma.** *Nat Med* 2002, **8:**816-824.

26. Yang XJ, Tan MH, Kim HL, Ditlev JA, Betten MW, Png CE, Kort EJ, Futami K, Furge KA, Takahashi M, et al: **A molecular classification of papillary renal cell carcinoma.** *Cancer Res* 2005, **65:**5628-5637.

27. Zhao H, Ljungberg B, Grankvist K, Rasmuson T, Tibshirani R, Brooks JD: **Gene expression profiling predicts survival in conventional renal cell carcinoma.** *PLoS Med* 2006, **3:**e13.

28. Zhan F, Huang Y, Colla S, Stewart JP, Hanamura I, Gupta S, Epstein J, Yaccoby S, Sawyer J, Burington B, et al: **The molecular classification of multiple myeloma.** *Blood* 2006, **108:**2020-2028.

29. Carrasco DR, Tonon G, Huang Y, Zhang Y, Sinha R, Feng B, Stewart JP, Zhan F, Khatry D, Protopopova M, et al: **High-resolution genomic profiles define distinct clinico-pathogenetic subgroups of multiple myeloma patients.** *Cancer Cell* 2006, **9:**313-325.

30. Tothill RW, Tinker AV, George J, Brown R, Fox SB, Lade S, Johnson DS, Trivett MK, Etemadmoghadam D, Locandro B, et al: **Novel molecular subtypes of serous and endometrioid ovarian cancer linked to clinical outcome.** *Clin Cancer Res* 2008, **14:**5198-5208.

31. Lu KH, Patterson AP, Wang L, Marquez RT, Atkinson EN, Baggerly KA, Ramoth LR, Rosen DG, Liu J, Hellstrom I, et al: **Selection of potential markers for epithelial ovarian cancer with gene expression arrays and recursive descent partition analysis.** *Clin Cancer Res* 2004, **10:**3291-3300.

32. Nakagawa T, Kollmeyer TM, Morlan BW, Anderson SK, Bergstralh EJ, Davis BJ, Asmann YW, Klee GG, Ballman KV, Jenkins RB: **A tissue biomarker panel predicting systemic progression after PSA recurrence post-definitive prostate cancer therapy.** *PLoS One* 2008, **3:**e2318.

33. Chen X, Leung SY, Yuen ST, Chu KM, Ji J, Li R, Chan AS, Law S, Troyanskaya OG, Wong J, et al: **Variation in gene expression patterns in human gastric cancers.** *Mol Biol Cell* 2003, **14:**3208-3215.

34. Cromer A, Carles A, Millon R, Ganguli G, Chalmel F, Lemaire F, Young J, Dembele D, Thibault C, Muller D, et al: **Identification of genes associated with tumorigenesis and metastatic potential of hypopharyngeal cancer by microarray analysis.** *Oncogene* 2004, **23:**2484-2498.

35. Chung CH, Parker JS, Karaca G, Wu J, Funkhouser WK, Moore D, Butterfoss D, Xiang D, Zanation A, Yin X, et al: **Molecular classification of head and neck squamous cell carcinomas using patterns of gene expression.** *Cancer Cell* 2004, **5:**489-500.

36. Lenz G, Wright G, Dave SS, Xiao W, Powell J, Zhao H, Xu W, Tan B, Goldschmidt N, Iqbal J, et al: **Stromal gene signatures in large-B-cell lymphomas.** *N Engl J Med* 2008, **359:**2313-2323.

37. Garber ME, Troyanskaya OG, Schluens K, Petersen S, Thaesler Z, Pacyna-Gengelbach M, van de Rijn M, Rosen GD, Perou CM, Whyte RI, et al: **Diversity of gene expression in adenocarcinoma of the lung.** *Proc Natl Acad Sci U S A* 2001, **98:**13784-13789.
