# Supplementary material for: Gene expression profiling of mouse p53-deficient epidermal carcinoma defines molecular determinants of human cancer malignancy
Source: Mol Cancer. 2010 Jul 14;9:193. doi: 10.1186/1476-4598-9-193 (PMC2913987; doi:10.1186/1476-4598-9-193)
Supplement: Additional file 5 — Overlapping between underexpressed genes in the tumor signature of p53-deficient mouse and genes underexpressed in human primary tumors with malignant behavior. The figure represents the overlapping significance with signatures of poor-outcome human tumors from different anatomical locations (A) and the list of genes which are underexpressed in the majority of the human signatures (B). [file 1476-4598-9-193-S5.DOC]

**Addtional file 5.**

(A) Overlapping between underexpressed genes in the mouse p53-tumor signature and genes underexpressed in human primary tumors with malignant behavior from 5 different cancer types. n: number of human tumors analyzed. Bar plots represent the significance of the overlap between mouse p53-tumors and human tumor samples (p-val, Fisher’s exact test). Above the plot is represented the color codes for each cancer tissue.

(B) Common genes underexpressed in the malignant signature analysis. Mouse p53-tumor genes which are overexpressed in at list 4 out of 7 human malignant cancers are shown. These genes represent a mouse p53-tumor DOWN gene SIGNATURE in malignant human cancer
